# Design, New Materials, and Production Challenges of Bioplastics-Based Food Packaging

**DOI:** 10.3390/ma18030673

**Published:** 2025-02-03

**Authors:** Phil Rosenow, Carmen Fernández-Ayuso, Pedro López-García, Luis Francisco Minguez-Enkovaara

**Affiliations:** 1 Fraunhofer Institute for Process Engineering and Packaging IVV, 85354 Freising, Germany; phil.rosenow@ivv.fraunhofer.de; 2 Centro Tecnológico Del Calzado y Del Plástico De La Región De Murcia (CETEC), 30840 Alhama de Murcia, Spain; c.fernandez@ctcalzado.org (C.F.-A.); p.lopez@ctcalzado.org (P.L.-G.)

**Keywords:** food packaging, bioplastics, packaging design, sustainable packaging, packaging processing, development of packaging materials

## Abstract

This paper outlines the current design trends in food packaging, its main environmentally friendly material alternatives, and industrial processing technologies. In this respect, this important product has undergone several evolutions throughout history. Initially acting as a containment device, it has later evolved into a source of information and even a marketing platform for food companies, always with a view to extending shelf life. However, these functionalities are highly dependent on the materials used and their properties. In this respect, plastics have conquered the food packaging market due to their affordability and flexibility. Nevertheless, environmental concerns have arisen due to their impact on the environment, in addition to the introduction of stricter industry regulations and increased consumer environmental awareness. Therefore, this work found that the current design trends in food packaging are toward sustainability, reducing packaging complexity, with easier recycling, and material selection that combines both sustainability and functionality. In the case of bioplastics as a sustainable alternative, there is still room for improvement in their production, with careful consideration of their raw materials. In addition, their technical performance is generally lower, with challenges in barrier properties and processability, which could be addressed with the adoption of Industry 4.0.

## 1. Introduction

Food packaging can be considered as a product that fulfils several requirements, primarily to provide consumers with reliable food quality in a cost-effective manner. However, its historical evolution has led to the additional provision of containment and convenience for the consumer, information about its contents, a marketing surface for the stakeholder to build brand identity and attract consumer attention [1], and protection of the packaged food against contamination and external influences, thus keeping it safe and extending its shelf life [2,3]. Over the years, the transport of food, for example, for its distribution or international export, has increased significantly, and has become a key pillar of many countries’ economies. As a result, the need for efficient food packaging and timely delivery to supermarkets has grown significantly. However, during the various stages of handling, transportation, and storage, food products are exposed to conditions that can lead to dehydration, spoilage, and a decline in visual appeal, flavour, and, most critically, nutritional value.

After its use, the packaging is often discarded and recycled, incinerated, or disposed of in landfills. In the worst case, it may end up in the environment. Packaging design needs to address all these factors: protective function, consumer interaction, and end-of-life [4]. The design of a food packaging unit includes a complex melange of different demands and tools, such as the selection of materials or the structural design or the graphical design of the packaging. Apart from the functional properties of the product, it still needs to be produced, with the resulting increase in its carbon footprint. Furthermore, nowadays, there is an awareness for the environmental impact of such a product resulting in stricter regulations and consumer’s environmental demands, pushing from the established linear to a circular economy [5,6].

The abovementioned is dependent on material selection, which has been subjected to several evolutions in our history. All of these are close knitted to changes in our culture and other related factors from population concentration in communities and cities to industrial revolutions. Nowadays, the need to overcome the climate emergency due to the pollution of the environment or the depletion of resources is leading to new industrial trends [7,8].

An example of primary materials for food packaging could be pottery, which was widely used in ancient civilisations. Subsequently, the first industrial revolution in the 18th century led to major improvements in this area with the development of the wood, paper, metal, and glass industries. More recently, the 1950s saw a boom in plastic food packaging, followed by major packaging technology breakthroughs [7,8,9]. Examples include aseptic processing and packaging, modified atmosphere packaging, or active and intelligent packaging systems.

In addition, food packaging technologies and materials have developed in parallel throughout history. One example is the First Industrial Revolution, when developments in ironworking led to the first tinplate cans, which helped various armies and British expeditions preserve their food supplies. However, these early periods also saw examples of food poisoning caused by the lead used to solder can lids. This was solved by other design alternatives such as glass containers, which proved to be an inert packaging solution with extended preservation properties compared to steel. The latter’s inorganic composition and amorphous structure give it properties such as rigidity, clarity, and inertness, but also weight and fragility. However, it was not until the early 1900s that their industrial production became cost-effective [7,8,9].

On the other hand, the paper industry exploded in the late 1800s, and this organic material became a viable solution for industries such as food packaging. These plant cellulose-based products are inert and environmentally friendly. However, they provide a weak barrier to moisture, and include formats such as sheets for wrapping and bags; moulded pulp for egg containers; or the more recent beverage containers in multilayered films. Furthermore, it is also worth noting that coated or multilayer paper products have become popular, as a solution to the above disadvantages. A prominent example of this is the multilayered structure marketed as Pure Pak in the US, and later as Tetra Pak in Europe [7,8,9].

More recently, aluminium has gained interest in bottle and film formats. This is due to its resistance to moisture, air, and microorganisms and its excellent formability. Nevertheless, its use as a food packaging material has been less widespread compared to the popularity of plastics after their boom in the 1950s [7,8,9].

Since then, plastics have overtaken other material alternatives by providing a good barrier for perishable products with the added benefit of a reduced weight. They are also easy to process and, with special techniques and additives, can become as good a barrier as metal or glass.

This type of material can be processed into the different formats required for the specific needs of food packaging, mainly films, trays, jars, or bottles. As a result, there are various production routes and equipment available on the market, designed to provide a good mix of materials and the appropriate shape. However, stricter regulations and more environmentally conscious consumer demands are gradually replacing oil-based plastics with more sustainable alternatives. The latter can be biobased, biodegradable, or have a high recycled content. Nevertheless, they are generally more expensive, more sensitive to the processing conditions, and have lower technical performance [10].

The following review summarises the state of the art of sustainable food packaging mainly based on bioplastic materials, focusing on the innovations that are emerging and other technologies that may be introduced in the coming years.

**Figure 1:**
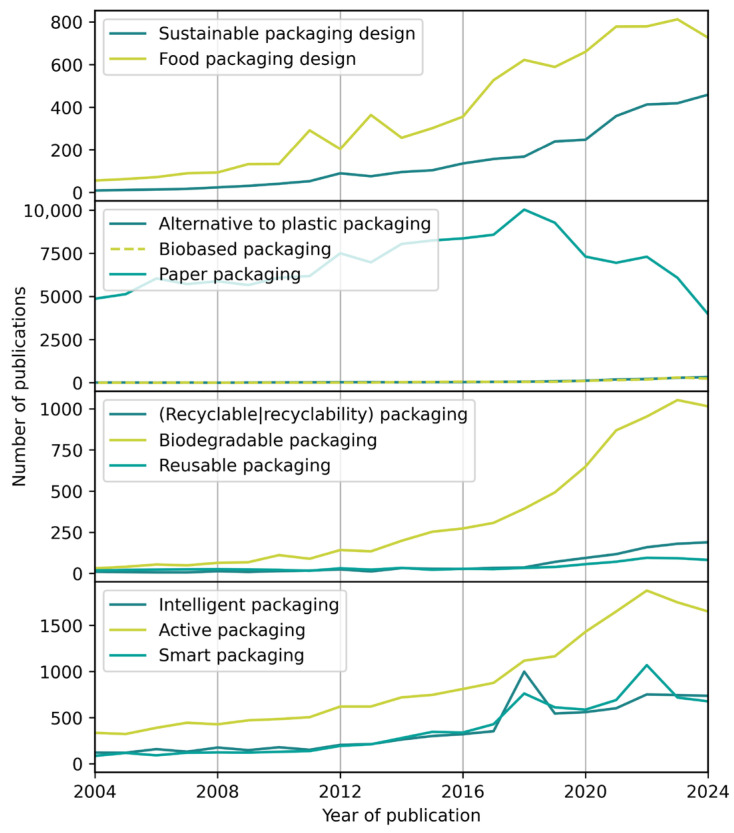
The number of publications on queried phrases according to Web of Science. The union of “recyclable packaging” and “recyclability packaging” is a combination of two independent queries.

To gauge the development of interest in different packaging design trends, the number of publications per year on a series of terms was queried from Web of Science for the last twenty years, as described in Section 2. The results are shown in Figure 1 and will be briefly discussed in the following. Both the terms “Sustainable packaging design” and “food packaging design” have a distinct growth in the timeframe in question, with the latter generally higher than the former. It is noteworthy that the number of publications for “sustainable packaging design” shows a more pronounced growth over the last five years, highlighting the increased importance placed on sustainability.

Since plastic packaging is often regarded as harmful in the wider public, the term “Alternative to plastic packaging” was searched and is compared to “biobased packaging” and “paper packaging”. The number of publications on paper packaging far outshines the other two searches but shows a marked downturn since 2018. This may indicate that—at least in the scientific community—the perceived trend toward paper shown in the general public has reached its peak. Alternatives to plastic and biobased packaging have been growing slowly but steadily in publications over the last two decades but are still far below the number of publications on paper packaging (which, of course, may also incorporate transport packaging).

Given the importance of the end-of-life of packaging, both in terms of public perception and environmental impact, searches were conducted for “Recyclable packaging” and “Recyclability packaging” (the results were combined into one) as well as “Biodegradable packaging” and “Reusable packaging”. Publications on biodegradable packaging show a pronounced rise in numbers starting in the early 2010s, outweighing the publications on recyclable and reusable packaging. Both these terms show an upturn in publications starting 2018, with the research output on recyclable packaging being slightly higher than on reusable packaging. While this may indicate that the interest in biodegradable packaging is higher than that in recyclable or reusable packaging, it should be remembered that both recyclable and reusable packaging are more established and available in the market and thus provide a less enticing field of research.

Finally, searches for “Intelligent packaging”, “Active packaging”, and “Smart packaging” were included to account for innovative features in these regards. The research output for all three terms has been increasing over the last twenty years, with more publications on active packaging than on intelligent or smart packaging.

Taking a bird’s eye view, even with its sharp decline, paper packaging is still the most published term within the search presented here. However, it is the only search with the number of publications in 2024 being lower than in 2004. Biodegradable packaging shows the strongest increase in the number of publications, making it a clear trend in scientific research on packaging design. While biodegradable packaging surpasses biobased packaging by a factor of four in the number of publications in 2024, these two aspects often go hand in hand. The observed discrepancy may indicate a priority in end-of-life over origin as a driver of scientific research.

## 2. Methodology

The aim of this review was to establish the state of the art of new design and manufacturing trends for food packaging based on sustainable bioplastics. For this reason, the review was divided into three main topics: food packaging design trends, emerging bioplastics that can be used in food packaging, and the main processing techniques available for food packaging based on these materials.

To do this, recent publications were prioritised, mostly between 2020 and 2024. In addition, the search was performed using the following search engines: Google, Google Scholar, Web of Science, and Elsevier.

For the search, the following categories and their related keywords were used:

- Design trends. Food packaging design trends, sustainable packaging design, innovations packaging design, Food packaging ergonomics, Food packaging user experience, food packaging ease of use, reusable food packaging, food packaging sustainability consumer, packaging free shopping, food packaging barrier innovations.
  In the case of Figure 1, it is based on the results of a bibliographic search carried out in Web of Science. For the latter, a timeframe between 2004 and 2024 and the following phrases were defined: “Sustainable packaging design”, “Food packaging design”, “Alternative to plastic packaging”, “Biobased packaging”, “Paper packaging”, “Recyclable packaging” and “recyclability packaging” (these two searches were combined), “Biodegradable packaging”, “Reusable packaging”, “Intelligent packaging”, “Active packaging”, “Smart packaging”.
- Food packaging materials. Feedstocks (biobased, algae, lignocellulosic), food packaging, food packaging materials, biobased materials, biodegradable materials, bioplastics, sustainable materials, PHA, PLA, TPS, barrier properties, plastics recycling, mechanical recycling, chemical recycling.
- Smart and intelligent packaging. Smart (food) packaging, Intelligent (food) packaging.
- Manufacturing routes and innovations. Extrusion, injection moulding, blown film extrusion, thermoforming, rotational moulding, additive manufacturing, laser developments, microwave heating, ultrasound, plasma, electrospinning, surface grafting, industry 4.0, industry 5.0, digital twins, machine aided learning, decision support systems.

## 3. Contemporary Approaches to Food Packaging Design

Packaging design encompasses the process of laying out packaging for a specific purpose and must consider all aspects of the packaging from production to end-of-life. This includes the protective function of the packaging, marketing and information aspects, consumer interaction, and disposal. Pressures on the dominant plastics packaging mount due to the environmental impact of plastic packaging waste, e.g., in the form of the Packaging and Packaging Waste Directive (PPWD) in the EU [11]. As such, packaging design innovations are essential for the transformation to more sustainable packaging strategies.

In this context, some terms shall be defined as follows:

- Sustainable packaging: The Sustainable Packaging Coalition outlines five criteria for sustainable packaging [12]. The first three criteria apply to the packaging itself and stipulate that it “uses SMART design” (system approach, material health, accessibility, reduction and elimination, lifecycle thinking), “advances the use of recycled materials and/or sustainably-sourced, renewable feedstocks”, and “is designed for reusability, recyclability, or compostability and labeled with appropriate end-of-life instructions”. The last two criteria stipulate that the company producing the sustainable packaging “engages with reuse and refill models” and “invests in the growth of recycling and composting infrastructure, collection and access”.
- Recyclable materials: Materials are considered recyclable when it is technically possible and economically viable to transform disposed material into usable materials and when this process is carried out in practice. This can include any available recycling method (mechanical-, chemical-, and solvent-based).
- Biodegradable materials: Materials are considered biodegradable when they decompose through biological means into non-harmful components within a given timeframe. Different conditions may be applied (industrial composting, home composting, different natural environments). Degradation into microparticles without further chemical decomposition is not sufficient.
- Reusable packaging: Reusable packaging encompasses all types of packaging intended to be used repeatedly. This includes returnable packaging to be collected, cleaned, and refilled by service providers and refillable packaging to be refilled at designated stations by the consumer.

Finally, it should be stressed that truly sustainable design requires a holistic view of the packaging value chain, including environmental, economic, and social factors, while addressing the actual demands and problems related to the packaged product. The whole life cycle of packaging and product must be accounted for, i.e., from production via distribution and consumption to end-of-life. Likewise, it is not sufficient to consider the product alone: related services and functions of the packaging must be included in the design process. To ensure a successful design, it is also advisable to include experts, stakeholders, and end-users in the process [13,14]. This melange of different factors inadvertently leads to a highly complex set of demands for research, development, and innovation, which necessitates prioritisation according to the available resources, timeframe, and scope. Decision support tools and metrics to aid in the packaging design are an active field of development [15,16].

Effective packaging design can contribute to the United Nations’ Sustainable Development Goals (SDGs) by prolonging the shelf life of packaged food, thus reducing food waste (SDG 2—zero hunger), reducing the incidence of food borne diseases due to improper storage as well as the use of food safe materials (SDG 3—good health and well-being), providing opportunities for employment, industrial development and innovation (SDG 9—industry, innovation and infrastructure), and reducing climate impact through reduced food waste and use of sustainable materials (SDG 13—climate action).

Trends related to different aspects of packaging design will be discussed in the remainder of this section.

### 3.1. Consumer-Oriented and Ergonomic Design

Ergonomics is the study of the interaction between humans and other elements, focused on the optimisation of human well-being and overall performance. In packaging, ergonomics mostly affects the ease of handling and opening of a given package, specifically including people with physical limitations, e.g., caused by conditions such as rheumatoid arthritis or osteoarthritis.

As highlighted by Bošnjaković and Vladić, assessing the ergonomics of packaging products requires a multifaceted approach, combining mechanical methods (measuring the force required to perform a given task), subjective methods (questionnaires related to the felt discomfort when performing a task), objective methods (a combination of subjective and mechanical methods), and usability methods (assessing a product during its use combining subjective and objective methods) [17]. A package should be easy to open for the customer, while at the same time be hard enough to open to prevent theft and sturdy enough to resist accidental opening during transport and handling. The ergonomics of opening a package are determined by a variety of factors: the visibility and clarity of the opening mechanism, its position relative to the holding position, the strength required to open, the required use of both hands, the required grip, the tightness of the opening mechanism, the fragility of the opening mechanism and the packaging itself, the slipperiness of the material, and the retention of the product upon opening. At the same time, the packaging must be able to be closed again if the contents are not intended to be consumed in one go.

The impact of arthritis on the opening process can be approximated using the Cambridge Simulation Glove (CSG), as performed by Reese et al. on five different products (soup can, a frozen rice product, string cheese, chocolate truffles, and a pill bottle) [18]. The bag of chocolate truffles provided minor difficulty due to the slipperiness of the glossy packaging, the strength of the sealing, and the small size of the gripping area. The primary packaging of the individual string cheese sticks, the shrink film around the lid of the frozen rice product packaging, and the opening tab of the soup can provided difficulty due to the tightness of the respective packaging, e.g., the proximity of the opening tab of the can. These observations indicate that sufficient space to operate the opening mechanism on a packaging unit should be included in the design of packaging to alleviate difficulties for people with impaired dexterity. The most difficult to open packaging in the study was the tamper-evident seal of the pill bottle, which required the user to apply force to very small tabs. A redesign performed in the study suggested to include a finger loop to make the opening easier for people suffering from arthritis.

As an alternative to integrating an easier opening mechanism into the packaging itself, it is possible to use assistant openers as external tools. Here, a combination of different opening mechanisms into one tool can provide aid to people with impaired dexterity [19].

Apart from dexterity, visual impairment, which may affect elderly consumers in particular, can impact how a consumer interacts with packaging, both in terms of recognition of information printed on the packaging and in terms of handling. A study by Plečko et al. suggests that using easy opening mechanisms, such as sufficiently sized lids, a surface textured to enhance the grip, and breakage-resistant materials (e.g., plastic rather than glass) provide benefits to consumers with impaired vision and reduced motor skills [20]. The use of solid colours rather than transparent packaging increases the visibility of the package to visually impaired consumers. The use of well-chosen colours, contrasting colours, and large enough fonts also helps this group of customers.

The ease of opening for adults is contrasted in packaging for products like medication and cleaning supplies by the necessity of preventing children from opening the package and gaining access to the contents using child-resistant closures (CRCs). Different studies show that children use a limited number of grip strategies to open CRCs compared to adults [21], and that adults aged 65 and older can exert more torque than children under 5 years old [22]. The latter study also suggested that limiting the functional surface area helps to bridge the gap between child resistance and ease of opening for geriatric adults. Numerical models can be used to make predictions on the opening characteristics of CRCs and thus have potential to be used in design optimisations [23].

The emptiability of packaging plays a significant role both in the user experience when handling the product, but also the sustainability of the package when it comes to preventing food waste [24]. Design choices in the shape and handling of the packaging influence the emptiability, depending on the packaged product, and may favour specific handling, which needs to be communicated to the consumer [25]. In addition, increasing the hydrophobicity of the packaging’s inside can make it easier to empty packaging, which can be achieved by applying hydrophobic coatings, such as waxes, clays, and cellulose derivatives [26].

### 3.2. Role of Design in Sustainability and Environmental Impact

The environmental impact of packaging is determined at the design stage [4,27]. Design choices can facilitate or hinder the sustainability of the final product, which in the context of sustainability assessment is not just the packaging itself, but the combination of packaging and packaged good, considering the entire production chain [28]. Two potentially contrary factors come into play here: on one hand, a minimalistic packaging has a lower environmental footprint by itself and is more suitable for recycling; on the other hand, the functional properties of the packaging for the protection of the product must be guaranteed [2]. This is especially the case for resource-intensive products. Since the sustainability of packaging is strongly affected by its end-of-life and by consumer behaviour, these aspects must also be considered at the design stage [27].

#### 3.2.1. Material Selection

The selection of materials and material combinations is a crucial part of sustainable packaging design. In recent years, there has been a push to replace complex, non-recyclable materials with monomaterials with comparable protective properties where possible [29]. The actual recyclability is also determined by the availability of recycling streams for the chosen material, which in turn requires the respective material to be technically and economically recyclable. These factors depend on the material’s ability to withstand the chosen recycling process, the abundance of the material in circulation, and the cost of the full recycling process in comparison to the production of virgin material. A driving factor for changing material selection in the EU is the Packaging and Packaging Waste Directive (PPWD), which stipulates increasing recycling targets for different packaging categories to be reached by 2025 and 2030 [11].

**Figure 2:**
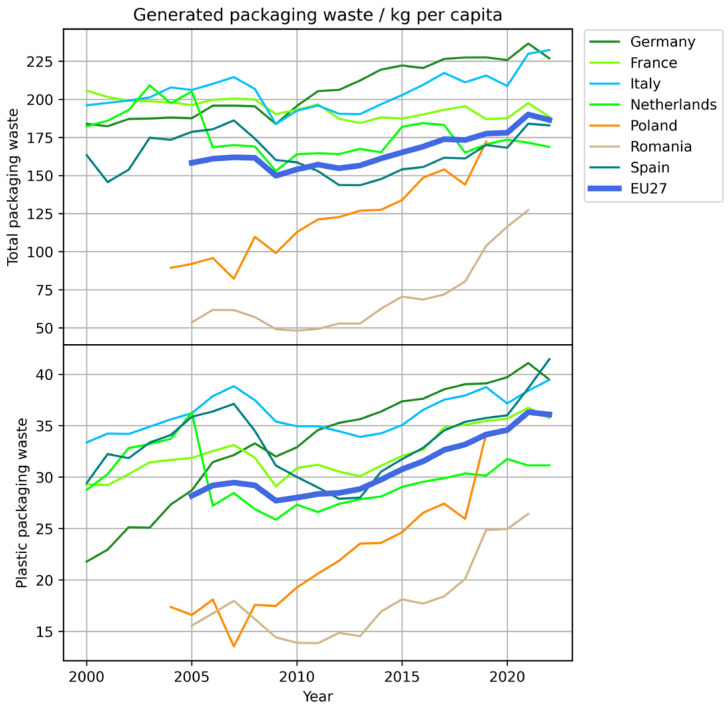
The generated total (**top**) and plastic (**bottom**) packaging waste in kg per capita across the EU and selected member states from 2000 to 2022. The countries selected each have a population of more than 15 million and comprise 74% of the total population of the EU. The figure was generated from Eurostat data [30].

**Figure 3:**
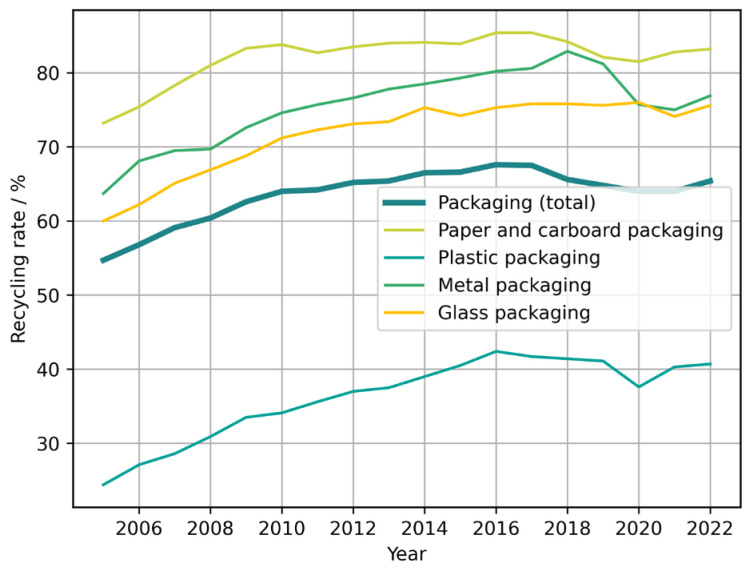
Recycling rates for different packaging materials in EU27. Figure generated from Eurostat data [31].

According to Eurostat, inhabitants of the EU generated around 190 kg per capita of total packaging waste (for all materials and types of packaged goods) in 2022, with strong variations among the different countries contributing to the statistics [30]. The amount of generated packaging waste has generally increased over the last 20 years (cf. Figure 2). The generated plastic packaging waste amounts to approximately 36 kg per capita, or less of a fifth of the total packaging waste by mass. Since plastic packaging is lighter than most other forms and the total packaging encompasses transport packaging as well, the role of plastic packaging is clearly significant. As shown in Figure 3, the recycling rate for plastic packaging, though improved in the last 20 years, is also lower than that for other types of packaging materials, which exacerbates its impact after disposal [31]. Again, this figure encompasses packaging for any use, not just food.

**Figure 4:**
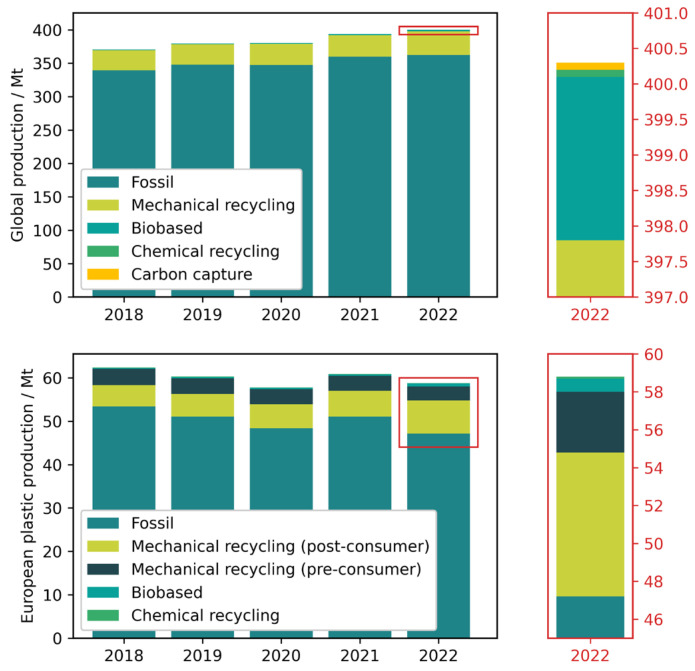
Global (**top**) and European (EU27+3, (**bottom**)) plastics production by feedstock. The right inset highlights the smaller contributors for 2022. The figure was generated from data published in the fast Facts by Plastics Europe [32].

Plastics Europe reports that the global plastics production (total, not just for packaging) is dominated by fossil-based plastics, with mechanical recycling of post-consumer waste accounting for roughly 10% of the production of fossil-based virgin materials [32]. Biobased plastics only account for a small part of the total global production, with chemical recycling and carbon capture presently being marginal sources for plastic materials (see Figure 4). For Europe (EU27 + 3), fossil-based virgin material accounts for roughly 80% of the plastic production, with mechanical recycling of pre- and post-consumer waste making up most of the remainder. Biobased plastics account for a small, slowly growing part of the production, while chemical recycling at the current state is negligible. It is estimated, that 39% of European plastic production is used for packaging. These data do not allow for a derivation of specific material trends for (food) packaging. However, fossil plastics are the dominating material class for plastics, in general, and for food packaging. The relatively large fraction of mechanically recycled plastics in European production are not suitable for food packaging due to legislation, at least not without functional barriers. The production of bioplastics, which are suitable for food packaging, is increasing slowly. Chemical recycling is still a minor contributor to plastic production but may open new avenues of recyclate use in packaging.

#### 3.2.2. Recycling Considerations

The end-of-life stage must be considered in the design stage of a packaging product [27]. Recycling is an important and desired pathway for the end-of-life of packaging and should be facilitated by the packaging design when it is the intended one. Important factors in the recycling process include sorting both at the consumer and facility stage and the technical recyclability of the material.

The separation of different material streams is a crucial step in the recycling process. The first step of sorting occurs at the household level with sorting into different bins. Here, it is imperative to support the consumer in correct sorting through indications of the packaging: correct sorting should be made obvious by the design of the packaging—clear labels were shown to be an effective tool, though the meaning of the labels must be clear to the consumer to have the desired effect [33]. At the same time, sorting should not be easy to perform, but should not unnecessarily inconvenience the consumer [34]. It was also shown that more valuable seeming packaging tends to be sorted into recycling correctly, while packaging that appears cheap may be incorrectly sorted [35]. Overall, consumer behaviour must be taken into account during the design phase to facilitate correct sorting behaviour at the household level [36].

The emptiability of packaging was identified as an important factor in sorting behaviour [37]. How easily packaging can be emptied is affected by its design, specifically by the shape and by the seams of the packaging. In packaging that retains food residues after emptying, moulding can occur, which on one hand leads to minor food waste, while on the other hand, may introduce contaminants and potential off odours into the recyclate or, in the case of reusable packaging, impact the cleaning process.

After collection, sorting into material streams at the recycling facilities is performed in a series of steps, including gravity-based and floatation sorting. Frequently, infrared sensors are used for material identification, which can be hampered by certain colourants like carbon and metal particles [38]. Thus, the inclusion of these contents should be weighed at the design stage. Additional, emerging technologies include AI-based sorting via object recognition [39] and digital watermarks [40]. While promising, these technologies are not widespread and may thus be considered for future packaging design revisions.

To facilitate the recycling of packaging, it is advised to keep the number of different materials in a unit of packaging to a minimum [41]. In cases where the use of different materials is required, a modular design approach can ensure recyclability, provided it is easy and obvious for the customer to take apart the packaging [37,42]. The choice of barrier layers should be made so as not to hinder recyclability, e.g., favouring oxide layers (AlOx, SiOx) over metallisation or EVOH [29]. A plethora of design for recycling guidelines and recycling systems exists, e.g., the RecyClass guidelines for conventional plastics [43] and CEPI guidelines for paper-based packaging [44].

#### 3.2.3. Biobased Materials and Biodegradability

Biobased and biodegradable materials have been steadily gaining interest as alternatives to fossil polymers and for their potential to solve the issue of plastic remaining in the environment [45]. A potentially attractive feedstock for biopolymers comes in the form of waste streams, both from agricultural waste and food waste, as the utilisation of waste streams reduces the environmental cost of the decomposition of those wastes while at the same time providing a source of packaging materials [46]. Many varied sources of agro-food waste exist, as do possible applications beyond packaging [47,48]. A study on agro-food waste from the production of major crops in the UK by Bolaji et al. indicates that the seasonal and regional distribution of different feedstocks as well as competitive use (e.g., animal feed, soil enrichment) must be taken into account in the sourcing of biobased polymers [49]. Both harvesting waste (e.g., straws) and processing waste (e.g., peels) are promising feedstocks for biopolymer production, but availability differs. The availability of the feedstocks is the determining factor of the economic viability of waste stream valorisation according to a cost–benefit analysis performed by Tassinari et al., who also point out that fossil-based plastics are currently cheaper than biobased ones [50].

Regarding biodegradability, a distinction must be made between degradability in different conditions, industrial composting plants, home composting, and degradability in the environment, with possible pathways depending on the characteristics of the material [45]. As was shown for the most common biobased polymer, PLA, limited compostability can be enhanced by including enzymes in the polymer matrix to enhance its degradation under milder conditions [51]. This approach offers perspectives for the enhancement of the biodegradability of biobased polymers, with the potential to streamline industrial composting and biopolymer degradation.

#### 3.2.4. Reuseable Packaging

Reusable packaging offers an enticing alternative to disposable packaging. The sustainability of reusable packaging is strongly affected by the logistics of the collection, transport, and cleaning infrastructure—in a European context, LCAs (Life Cycle Assessments) tend to favour reusable packaging over single-use [52,53]. While these considerations go beyond the realm of packaging design, the latter can aid with logistical aspects by keeping transportability in mind during the design process, e.g., by choosing a shape that facilitates stacking [54].

From a design perspective, reusability poses its own requirements on packaging, such as a certain resistance to damages through use, e.g., scratching or breaking. The design of refillable packaging as a subset of reusable packaging should account for easy and obvious emptying, refilling, and cleanability [55].

For reusable packaging to be a success, it must be accepted by the consumer. Thus, it is important to identify factors that may help or hinder consumer participation. A clear communication on the safety of reusable packaging was shown to increase consumer willingness in a survey setting [56].

#### 3.2.5. Consumer Perceptions of Sustainability

Another crucial aspect of packaging design is communication to the consumer, relating to the perception of the product’s quality as well as the sustainability of the packaged product. Steenis et al. queried Dutch students on their perception of tomato soup packaged in seven different structural designs with two graphical schemes each [57]. The different structural schemes were assessed regarding their sustainability by the participants of the study and by LCAs. A stark discrepancy between perception and LCAs was found for most of the structural designs. For example, glass jars scored least sustainable in the LCA but were perceived as sustainable by the participants. On the other hand, dry carton sachets were rated most sustainable by the LCA and highly unsustainable by participants. For liquid cartons, the agreement between LCA and participant perception was best.

Boesen et al. performed a perception study with LCA comparison with young consumers in Denmark on different packaging types for beverages [58]. Apart from the perceived sustainability, this study also asked how the participants reached their conclusion. The participants mainly considered the material type and their disposal options in their assessment, while barely considering production and transport of the packaging. Glass bottles and biomaterial packaging were perceived as the most sustainable, laminated cartons received a mixed perception, and plastic packaging was perceived as the least sustainable. In contrast, LCA showed plastic and laminated cartons as the more sustainable options, despite limitations when it comes to recycling. A study by De Feo et al. surveying Italian students came to similar conclusions, showing that the participants perceived single-use glass bottles as more sustainable than plastic bottles, with LCA data indicating the opposite [59].

A survey conducted by Herbes et al. with participants from France, Germany, and the USA came to similar results on the decision factors of consumers: again, material and disposal were the most important factors for consumers, whereas production, transportation, and use were less considered [60]. The study also found that material reduction, recyclability, biodegradability, reusability, and the use of recyclate were identified as environmentally friendly by the surveyed consumers. In another study by Herbes et al., the importance of different cues to the consumers’ perception of sustainability was surveyed, again with participants from France, Germany, and the USA [61]. Labels ranked highly with consumers from all countries. French participants also considered the material on par with the label. Both German and US participants replied with receiving further information as an important decision factor, followed by information on the packaging. Among materials, paper was generally regarded as more sustainable, while plastic was not mentioned as favourable by consumers in any of the surveyed countries.

These findings on consumer perceptions and behaviours have implications beyond packaging design and highlight the importance of consumer information.

#### 3.2.6. Packaging-Free Shopping

Another approach to sustainable shopping is the relinquishment of primary packaging altogether, at least for food products that do not require strong protection and can be acquired in customer-brought containers. Gordon-Wilson et al. showed, in a survey among British consumers, that the decision for packaging-free products is largely driven by green consumption values rather than value consciousness and is part of a lifestyle, not just shopping behaviour [62]. The shoppers who prefer packaging-free shopping also put a considerable effort in the planning and preparation of their shopping trips. This study indicates that packaging-free shopping may not be a mass solution but be favoured by more affluent consumers with sufficient time. A study among Portuguese customers by Barbosa et al. showed that packaging-free products can positively affect brand image, brand trust, and customer loyalty and satisfaction [63]. Similarly, Louis et al. found that offering packaging-free products can tighten the relationship between stores and customers [64].

### 3.3. Packaging Materials Development for Functional Design

Protecting the product from contamination and extending its shelf life are the main functions of food packaging. The product protection requirements imposed on the packaging depend strongly on the packaged product and should be tailored to the same. Oxygen and water vapour barriers, transparency, and mechanical protection are commonly accounted for in the design process by choosing materials with suitable properties. Mechanical protection in particular is also affected by the structural design.

High barrier requirements have traditionally been satisfied with complex multilayer materials, which provide barriers to recycling and, for fossil-based materials, biodegradability. Legally, multi-materials are defined by the mass of the different materials, making thin barrier layers a viable option to replace conventional compounds [65]. Water-based biopolymers and nano-biocomposites are among the material classes under heavy development due to their potential for eco-friendly application [66,67] but are still overshadowed in their prevalence by fossil-based materials, as discussed in Section 3.2.1. Reduction in complexity and material use by innovative design are noteworthy trends on the market, as will be showcased in Section 3.5. Some recent innovations for biodegradable barrier lacquers, paper coatings, and liners for metal packaging will be discussed in the following.

By choosing the right composition, barrier lacquers can be made to be biodegradable, thus neither hindering recycling due to their low content nor preventing biodegradability. For example, a compostable functional barrier coating based on ORMOCER^®^ (Fraunhofer ISC, Würzburg, Germany), dubbed bioORMOCER^®^, with added hemicellulose provides an oxygen barrier as a thin layer whilst being biodegradable [68]. Polyvinyl alcohol (PVA), a biodegradable material which is likewise used for its oxygen barrier, has recently increased in price. Functionalising it with montmorillonite nanoparticles allows for a high oxygen barrier when applied in thin layers via reverse gravure coating, thus performing its protective function while using less material [69].

In the same vein, the public perception of paper versus plastic as packaging material has driven interest in paper-based packaging [70]. Since paper alone provides poor barriers, its protective function must be established using coatings. While conventional fossil polymers are a viable and often used option, biobased coatings can be applied as well [71]. To reach very high barriers, metallisation is frequently applied, which is very challenging on paper. New procedures like transfer metallisation are under investigation, but not quite market ready [72].

Metal packaging (such as cans) requires a lining to prevent interaction between the content and the metal [73]. In this sector, too, biobased lacquers utilising waste streams are being investigated, e.g., based on lipids from tomato pomace as alternatives to Bisphenol A-containing linings [74]. These biobased lacquers have shown to be favourable in the LCA.

Advancements in user-friendly features of packaging design are called out in recommendations to packaging designers. The importance of accessibility is clearly mentioned (see also Section 3.1),e.g., in supplying easy opening mechanisms, clear labelling, and the possible inclusion of Braille labelling as well as the avoidance of information overload while offering audio information per QR code to keep information accessible for consumers with difficulties reading [75]. The clarity of information is likewise recognised as a way to improve user experience, especially when it comes to the display of allergens, shelf life, health benefits, and preparation instructions [76].

### 3.4. Influence of Consumer Preferences and Market Trends

Consumer preferences and market trends create incentives and pressures for companies, which may affect the design of their packaging. It is ultimately a consumer decision whether to buy a product or not. This decision can be influenced by the design of the packaging, especially when the shape, graphical design, or material is changed. Regarding sustainability, consumers show a preference to redesign favouring circular approaches over linear ones, with combinations of several design strategies not showing a significant effect [77].

Customers place importance on the protective features and safety of packaging [78]. The latter can be influenced, e.g., by the transparency of the packaging and thus the ability to visually assess the state of the packaged product. For foods with short shelf life, packaging was shown to be a major factor in the avoidance of food waste—a well-dimensioned package and the availability of clear instructions on how to safely store the package were shown to be highly relevant [2].

As discussed above, consumers who are conscious of a sustainable lifestyle pay attention to the packaging of the food they buy, mainly focusing on the source and end-of-life of the packaging. While this assessment misses important factors, it provides a pressure to packaging designers to acquiesce in their work [60,61]. In a study by Langley et al., participants valued the reduction in packaging higher than the avoidance of food waste [79]. The survey also indicates that food packaging is not the main driver in the purchasing decision for food, though the consumers’ perception on food packaging and its use to the consumer factor in.

Among the possible design choices, the sizing of the packaging related to the content stands out. From a functional perspective, the packaging should be well dimensioned to prevent both food and packaging waste [80]. On the European level, the Packaging and Packaging Waste Directive (PPWD) stipulates requirements on the filling level of a package, thus providing a pressure to reduce overpackaging in this regard.

The choice of packaging design and material may also affect the perceived value of the packaged good. If the packaging is perceived as cheap, this attribute may be transferred to the content by the consumer. Likewise, a packaging perceived as premium may elevate the value of the product in the consumer’s eyes. This can have adverse effects regarding sustainability if a reduction of overpackaging leads to a decrease in perceived value, which may negatively affect the purchasing decision, at least among non-environmentally conscious customers [81]. However, if the brand itself is perceived as premium, this effect can be mitigated [82].

Both the shape and colour of the packaging are closely tied to the brand identity and can thus influence the consumer’s decision to buy a product [83,84]. Creativity and uniqueness may help a packaging and its product to stand out [1].

Lastly, as the packaging is priced together with the product, changes in packaging material specifically can impact the retail price. The willingness of the consumer to pay for new packaging, potentially a more sustainable one, must be factored in when redesigning packaging. In surveys, customers sometimes show a willingness to pay an increased price for better packaging [78], but not always [85], which may be strongly influenced by the individual consumer’s attitude toward sustainability and their economic situation. However, it is not fully clear how this translates from surveys to actual consumption behaviour [86].

### 3.5. Case Studies of Successful Packaging Designs

To showcase successful design cases with high market applicability, the following examples were selected from winners of the German Packaging Award 2024 and 2023, awarded at the FachPack trade fair. The selected cases are summarised in Table 1.

The featured case studies showcase the importance of considering structural elements in the packaging design process. Significant reductions in material use could be achieved by replacing the amount of material with the use of stabilising (moulded tubs, bottles) or functional (cheese wrapper) elements. The use of monomaterials is likewise a common thread, taking the burden of component separation and sorting from both the consumer and the recycling system.

## 4. Emerging Bioplastics Tailored for Food Packaging

As aforementioned, in food packaging, there are several solutions for the selection of materials. However, plastics currently lead the market with their affordability and also their environmental concerns. They can be either thermoplastics or thermosets. The actual food packaging market extensively uses thermoplastics with the conventional plastic variants of polyethylene (PE), Polypropylene (PP), Polystyrene (PS), and polyethylene terephtalate (PET) [9]. These are historically synthesised from oil. However, as sustainability is imposing in the industry, their biobased counterparts are appearing, in parallel with other types of sustainable solutions.

Concerning them, several options were developed to reduce their environmental impact [94,95,96], such as the incorporation of additives to obtain oxo-biodegradable plastics, with later evidence of detrimental effects on the environment [97,98]. Or the more recent recycling processes, such as mechanical and chemical recycling. They can deliver downcycled or upcycled products, i.e., reprocessed products with a narrower or wider range of possibilities, respectively, compared to the virgin fossil reference [99,100]. However, they are still under development in need for further improvement of plastic sorting facilities and advances in chemical recycling technologies. All of these are pursuing the goal of a circular economy model [101,102].

In this respect, they appear to be a potentially viable solution to the current climate emergency, as far as the food packaging industry is concerned.

### 4.1. General Characteristics of Biopolymers

Bioplastics are a family of polymers with the dual capability of being made from natural or renewable resources and/or being biodegradable. This means that not necessarily all kinds of biopolymers are both biobased and biodegradable [103,104]. In this context, biobased refers to the origin of the raw materials used in the production of bioplastics. These are typically polysaccharides, lipids, proteins, woods, potatoes, corn, vegetable oil, cereal crops, or even food waste [105]. In addition, biobased bioplastics can distinguish, according to their origin, the categories of being directly extracted from biomass, produced from bio-derived intermediates, and produced by microorganisms. While biodegradability refers to the breakdown of the polymer chains of plastics, e.g., by microbes, which use them as a food source, or hydrolysis. However, the latter is considered to be highly dependent on the environment in which the polymers are disposed of, with different results for the same polymer. In this respect, the most aggressive is compost (industrial and domestic), followed by soil, fresh water, sea water, and finally landfill [106,107].

The aforementioned biobased plastics use biobased feedstocks that, with their advantages and disadvantages, appear to be environmentally friendly [108]. They are generally based on biomass, which can be derived from plant, crustacean, and animal sources. Plant biomass mainly provides starch, cellulose, hemicellulose, lignin, polyphenols, and triglycerides. On the other hand, chitin is obtained from crustaceans, and lastly, collagen, keratin proteins, or triglycerides are general animal products. In addition, starch and cellulose can be processed to produce glucose polysaccharides, while starch can also provide triglycerides in the form of oils [109]. All of these are generally classified according to their source of extraction, with the following classes being identified:

- First generation. This class represents edible agricultural products that can also be food resources for humans and animals. In this regard, this class can be controversial because it diverts supplies and resources needed for animal feed. However, it can also benefit farmers by providing them with alternative markets for their surplus production.
- Second generation. This class is characterised by the use of non-edible by-products, such as waste starch, cellulose, fatty acids, and organic waste from agricultural residues, or other streams such as biodegradable municipal waste or animal by-products. In this respect, there is a trend to develop this feedstock stream to address food availability concerns. In addition, this stream is in line with the goal of achieving a circular economy, where materials extend their life cycle by being used in different products.
- Third generation. It is attributed to algae that were previously considered as second generation, but which were distinguished as a different class due to their cultivation on the non-arable surface of the oceans. In this respect, algae, which can be micro- or macro-sized, have the potential to be grown and harvested in 70% of the Earth’s surface that is covered by the oceans.

In addition, feedstocks need to undergo transformation processes in order to become suitable raw materials for the production of bioplastics. To analyse this process, Moutsidi et al. [110] identified the main steps required to transform corn or other agricultural grains into usable raw materials for the chemical industry: crop cultivation, crop processing, fermentation, purification, and recovery.

Depending on their sources, bioplastics can be classified as shown in Table 2.

Apart from their origin, when selecting bioplastics with biodegradability, their reference environment of biodegradation needs also to be considered. This means that depending on the biopolymer, different waste management strategies at the end of their life cycle for their corresponding packaging need to be considered. Taking into account the results of Kim et al. [107], shown in Table 3, natural bioplastics such as those that are starch- or cellulose-based are biodegradable in all the reference environments. This is also the case of polyhydroxyalkanoates (PHAs). However, other aliphatic polyesters show this important feature under certain environment conditions, such as polylactic acid (PLA) or polycaprolactone (PCL). For this reason, sorting and waste management strategies should be taken into account to maximise the sustainable properties of the packaging materials.

In addition, bioplastics are generally differentiated in the following three main groups: biobased and biodegradable; biobased and non-biodegradable; and non-biobased and biodegradable.

#### 4.1.1. Biobased and Biodegradable Bioplastics

Biobased and biodegradable bioplastics stand nowadays as a win–win solution to the climate emergency. This is due to their wide range of bio-resources and their biodegradable nature. However, further development of cost-effective production strategies is needed. All this without diverting resources from human food production. The main examples of this category are polyhydroxyalkanoates (PHAs), polylactic acid (PLA), and thermoplastic starch (TPS), with the following attributes.

##### PHAs

The abovementioned origins of bioplastics demonstrate the potential contribution of biobased materials for the goal of a circular economy. Additionally, these biobased origins can imply a powerful upcycling solution where traditionally considered agricultural or food industry wastes can become a valuable raw material for the production of food packaging.

In this respect, PHAs represent a powerful bioplastics family due to their dual biobased origin and biodegradable properties. Furthermore, they stand out due to their biodegradability in all the reference scenarios. However, they are still more expensive than their fossil-based counterparts.

The PHAs are generally biosynthesised from up to 300 different microorganisms and provide similar properties to conventional plastics [117]. As a result, certain strains of microorganisms can be selected in order to produce them more efficiently. The latter depends on parameters such as the type of substrate used or other requirements necessary to control their biosynthesis. It is worth noting that the substrate is composed of elements such as oxygen, carbon, phosphorus, or nitrogen. The latter influencing the final cost of the polymer by about 50% [118]. Consequently, new strategies are being developed to use agricultural or food industry wastes as substrates, in order to turn the production cost-effective.

More than 150 variants of polyhydroxyalkanoates were reported, apart from their first ever discovery, Poly(3-hydroxybutirate) or PHB, in 1926 [119,120]. Since then, variants such as PHB, and copolymers poly(3-hydroxybutirate-co-3-hydroxyvalerate), poly(3hydroxybutirate-co-4-hydroxybutirate), or poly(3-hydroxybutirate-co-3-hydroxyhexanoate) have emerged as the most commercialised [121,122]. All these offer variable properties depending on parameters such as the type and content of the monomers combined [123]. An example of them is summarised, and compared to the conventional ones, in Table 4.

##### PLA

As mentioned above, polylactic acid (PLA) is a type of polyester with an aliphatic chain and thermoplastic nature. This is one of the most extended and widely used bioplastics in food packaging due to its high strength, processability, transparency, and compostability. It is also known for its dual biobased origin and biodegradable properties. As a result, it is currently considered as a substitute for many applications where conventional plastics were applied.

It exists in three different forms, such as poly(D-lactide), poly(L-lactide), and poly(D,L-lactide). The latter, characterised by its amorphous polymer nature, as opposed to the semicrystalline nature of the others [121]. Consequently, it has a chiral active polymer structure, and its properties can be easily modified depending on its constituents [122].

PLA can be derived from renewable resources such as sugarcane, maize, cassava, corn, etc.

However, its biodegradability is lower than, e.g., PHAs, being slow under mild conditions, preferably relying on industrial composting conditions.

##### Thermoplastic Starch (TPS)

It is worth noting starch as another promising biobased and biodegradable bioplastic. This is due to its renewable sustainable nature, being extracted from abundant and cost-effective resources, such as cassava, corn, or potato. The latter has a strong influence on the resulting material properties [126]. Furthermore, it is demonstrated to be biodegradable in all the reference conditions [103].

Starch consists of two glucose macromolecules, amylose and amylopectin, which can be synthesised in the form of native starch (NS) or thermoplastic starch (TPS). On the one hand, NS granules have to be plasticised in order to become deformable and thermoplastic. On the other hand, TPS, which is most commonly used as a food packaging material, is obtained by combining of NS with plasticisers, such as glycerol, urea, sorbitol, glycerin, or water [127,128]. However, their inherent properties prevent them from their direct use in food packaging. This is due to their high hydrophilicity and poor mechanical properties, such as low tensile strength. As a result, several structural alteration and chemical modification techniques were developed to meet the needs.

In this respect, TPS has an increased chain mobility, compared to NS, due to the applied plasticisers. As a result, it offers required flexibility, with the reduced brittleness and shrinkage needed to produce plastic films. However, it has inferior properties compared to its petroleum-based references. For this reason, TPS is commonly blended with other polymers in order to obtain suitable packaging materials.

#### 4.1.2. Biobased and Non-Biodegradable Bioplastics

Another category that is gaining interest among the industries is biobased conventional polymers. With their main attribute being their origin from renewable resources, instead of the petrochemical ones, maintaining their properties. As a result, they offer a reduced carbon footprint compared to their fossil-based references, on the one hand, while maintaining their slow biodegradability [129].

On the other side, the main concern for this class is their biobased content, which can vary between polymers, not always achieving 100%. Furthermore, this biobased origin may suppose a threat to the resources dedicated to human food production. For this reason, a careful life cycle assessment and the sustainability of their production should be assessed before mass production [130].

At present, the most renown polymers for this category are bio-PE, bio-PET, bio-PP, bio-polyamide (bio-PA), bio-poly (butylene succinate) (bio-PBS), and emerging variants such as bio-Ethylene2,5-Furandicarboxylate (bio-PEF).

#### 4.1.3. Non-Biobased and Biodegradable Bioplastics

Controversy may arise in relation to fossil-based bioplastics, which apart from their proven biodegradability, are synthesised from oil. This category includes well-known examples such as polyvinyl alcohol (PVA), polycaprolactone (PCL), Polybutylene adipate terephthalate (PBAT), poly (ethylene succinate) PES, poly (propylene succinate) PPS, or PBS, including its copolymer poly butylene succinate-co-adipate (PBSA). These can be used in the food packaging industry. In particular, they are often blended with others such as polylactic acid (PLA) or polyhydroxyalkanoates (PHAs), acting as plasticisers to reduce the inherent brittleness of the latter [103,131,132].

### 4.2. Properties and Strategies to Improve Bioplastic-Based Food Packaging

In parallel with the development of the plastics industry, various packaging technologies have flourished over the years to improve food preservation. These relate to specific designs and manufacturing processes applied to the packaging. They can also be based on principles ranging from the internal atmosphere of the container to the presence of certain substances or sensors, such as active additives [133]. In this respect, barrier properties play a key role in food packaging materials. In fact, they stand out among others like transparency, thermal resistance, or mechanical performance.

Barrier refers to the ability of the material to allow the exchange and permeation of low molecular weight chemical substances such as gases, water vapour, or certain organic vapours (or aromatic molecules), while, light, microbial, and oil barrier properties can also be considered.

All the aforementioned apply to food packaging due to the nature of the food, which is inherently chemically unstable. Therefore, it is of utmost importance to protect it from common deterioration situations such as, spoilage, lipid oxidation, or microbial contamination [134]. As a result, barrier properties can vary depending on the foods that need to be preserved, which is in line with the materials selected for the container, as the following explains.

- **Water vapour permeability (WVP)**. It determines the effectiveness of a material to prevent moisture loss for a particular type of food, such as in bakeryproducts. Currently, the most commonly used polymers in food packaging systems for the provision of water vapour barrier properties are LDPE and HDPE [135]. Water vapour permeability strongly depends on parameters such as the hydrophilicity or the surface roughness. In this respect, polymers with OH groups along their chemical structure, such as cellulose, chitosan, or starch, will provide a poor barrier to moisture. Furthermore, superhydrophobic surfaces will have a minimum contact angle of 150° and a sliding angle close to 10° [136].
- **Barrier capacity against molecular oxygen (O_2_)**. It is essential to guarantee the maximum shelf life of food products such as fats, proteins, pigments, vitamins, aromas, and flavours since oxygen is responsible for the oxidation reactions of these food components. Currently, the most widely used polymers as oxygen barrier materials are ethylene vinyl alcohol (EVOH), polyvinylidene chloride (PVDC), PET, and polyamide-6 (nylon), applied either as co-extruded or laminated films and coatings [137]. As a result, the polymer films used for food packaging are generally classified according to a range of oxygen and water vapour permeability values [138], as shown in Table 5.

**Table 5:** Scale of oxygen and water permeability for food packaging materials [138].

Barrier Grade	Oxygen Permeability (cc mil/m^2^ Day atm)	Oxygen Permeability Material Examples	Water Vapour Permeability (g mil/m^2^ Day kPa)	Water Vapour Permeability Material Examples
Very High	<1.6	EVOH, ~0.5 (23 °C/0%) [139] Aluminium ~0 [140]	<1.6	Aluminium ~0 [140]
High	1.6–16	PVOH ~3 (24 °C/0%) [139]	1.6–16	Oriented PP ~5–10 (23 °C/0%) [139] HDPE ~6 (40 °C/90%) [139]
Medium	16–160	Oriented PET ~35 (23 °C/0%) [139] Nylon 6 ~40 (23 °C/0%) [139]	16–40	EVOH ~33 (40 °C/90%) [139]
Low	160–1600	PBS ~208 (23 °C/50%) [141] 340 (20 °C 90%) [142]	40–120	PBS ~175 (25 °C) [143] PPC ~162 (23 °C/90%) [144] PBAT ~1380 (23 °C/100%) [145]
Poor	>1600	HDPE ~2325 (23 °C/0%) [139] PBAT ~2440 (23 °C/50%)) [145]	>120	PS ~132 (40 °C/90%) [139] Nylon 6 ~300 (37.8 °C/90%) [139]

PVOH: Poly (vinyl alcohol); HDPE: high density polyethylene; EVOH: Ethylene vinyl alcohol; PVDC: Poly(vinylidene chloride); PP: Polypropylene; PPC. Polypropylenecarbonate; PBAT: Polybutylene adipate terephtalate PBS: Polybutilsuccinate; temperature and relative humidity during measurement are provided in table.

- **Barrier properties against carbon dioxide (CO_2_)**. Since CO_2_ is generally added into the food packaging of some foods, e.g., fresh products, it allows for the preservation of their quality and even prolongs their shelf life by delaying microbial activity. Thus, keeping adequate levels of CO_2_ permeation is very relevant to prolonging shelf life [146].
- **Barrier properties against odours** from the outside. It is another important aspect to consider in food packaging materials since they might end up decreasing quality. Polymers that are currently applied efficiently for this purpose include PVDC, nylon, and PET.

Barrier properties are mainly based on the process of surface dissolution and subsequent diffusion through the thickness of the corresponding packaging material. The latter is generally described by Fick’s law [147]. Based on this theory, materials can be designed according to the preservation needs. As a result, in the case of different plastic packaging technologies requiring different gas barrier properties, it be distinguished as follows:

- Vacuum packaging aims to achieve the absence of gases, such as oxygen, inside a package. It is therefore used in the food industry to prevent the oxidation of oxygen-sensitive foods. As a result, it requires high gas barrier properties, combined with sufficient flexibility to prevent the collapse of the materials [148].
- Controlled atmosphere packaging consists of the introduction of a specific gas or mixture surrounding the perishable product. The modified atmosphere, which requires a specific gas permeability for O_2_, CO_2_, or N_2_, can operate in two ways. On the one hand, it can act passively, e.g., by delaying the growth of microorganisms. On the other hand, it can act actively by renewing the atmosphere with a controlled gas mixture [149].

Therefore, films in different materials show specific permeation rates, depending on parameters such as the chemical nature of their monomer constituents; the amorphous/crystalline ratio; the processing conditions; or other environmental factors such as temperature and humidity [150]. For example, when comparing the oxygen and carbon dioxide permeability of different materials, PLA shows higher permeability rates than PHBV, polyvinylidene chloride (PVDC), ethylene vinyl alcohol (EVOH), PET, and polyvinyl alcohol (PVOH), respectively [147,151].

For this reason, multilayer plastic films can be applied to achieve tailored atmosphere treatments. These can also be compounded to increase the functionality of the material. In fact, most conventional plastic packaging with barrier properties nowadays is based on multilayers (with each layer serving a specific function) and/or coatings. In this respect, a typical structure can be constituted by the outer print, moisture barrier, gas barrier, and heat sealable or food contact layers. In addition, these structures commonly include aluminium to ensure very high barrier properties [152,153,154].

The abovementioned layers can be processed using lamination, co-extrusion, compression moulding, electrohydrodynamic processing (e.g., electrospinning and electrospraying), or layer-by-layer deposition [155,156,157]. These strategies also include coating with synthetic polymer layers or reinforcement with nanomaterials. However, these food packaging structures may lead to detrimental effects in the recyclability and biodegradability at the end of their life cycle.

#### 4.2.1. Properties of Biodegradable Polymers

In recent years, research has focused on the development of innovative biobased and biodegradable packaging solutions for their application in the food industry to replace conventional fossil-based plastic packaging [157]. However, most biobased and biodegradable packaging materials lack suitable barrier properties to preserve food quality and freshness [134]. Below, the main examples of bioplastics are discussed.

##### PLA

PLA properties highly depend on stereochemistry, composition, and molecular weight. As a result, there are different PLA grades based on lactide (cyclic dimer of the chiral lactic acid moiety), which exists in three diastereoisomeric forms: L-lactide, D-lactide, and meso-lactide. Optically pure PLA, such as isotactic poly(L-lactide) (PLLA) and poly(D-lactide) (PDLA), is crystalline. While the atactic poly-(meso-lactide) (PDLLA) is completely amorphous. Therefore, according to the ratio of optically active L- and D, L-monomers, the amorphous and crystalline content of PLA can be tunned with its resulting properties. Also, there are other influencing factors such as chain orientation and crystal packing that can affect crystallinity with its content, crystal thickness, spherulite size, or morphology.

PLAs with very high content of amorphous regions and low molecular weight (MW) are not used in packaging as their thermal stability is lower and degrade more easily. Even the highest performing PLA grades with the highest degree of crystallinity, such as PLA with 96–99% L-lactide, are not good enough to extend the applicability of PLA in packaging. This is because of their relatively poor barrier performance to gases, such as oxygen, or even their poor heat resistance and brittle fracture behaviour, compared to oil-based counterparts [147].

##### PHA

Commercial PHAs like poly (3-hydroxybutyrate) (PHB) and its copolymers such as poly (3-hydroxybutyrate- co-3-hydroxyvalerate) (PHBV) or poly(3-hydroxybutyrate-co-4- hydroxybutyrate) (PHBH) show potential properties for their application into food packaging. This is mainly due to their good oxygen/water vapour barrier properties, with an oxygen permeability lower than 50 cc (gm).mil/m^2^-day-atm. This is the case of some PHBV grades with low hydroxyvalerate and high crystallinity, up to 60% [138]. As a result, the barrier properties decrease when increasing the hydroxyvalerate contents [158].

##### BioPBS

PBS is similar to PLA in terms of oxygen and water vapour barrier properties. The oxygen barrier properties of PBS with a crystallinity around ~35% are reported between 200 and 300 cc.mil/m^2^-day-atm, which is superior to PLA but inferior to PHB/PHBV. On the other hand, PBS is more flexible than PLA, which offers advantages for flexible packaging applications [138].

##### Natural Biopolymers

Natural biopolymers are long chain macromolecules made of repeating, covalently bonded units like polynucleotides, polypeptides, or polysaccharides.

In this respect, the abovementioned were demonstrated as potential sustainable moisture and oxygen barriers. These consist of polysaccharides, such as starch, cellulose, and chitosan; proteins, such as zein, whey, and gluten; or lipids, based on waxes. For example, nanocellulose incorporated into polymer matrixes is able to enhance properties such as tensile strength, barrier capacity, and antibacterial properties. In addition, nanocellulose can be used in functional coatings, such as those able to scavenge ethylene, and is able to extend the shelf life of fresh food by reducing the quantity of ethylene in the packing area [159].

On the other hand, thermoplastic starch (TPS) has good oxygen barrier properties. In fact, it was used as a reinforcement in combination with other biobased polymer matrixes such as PLA or PHA for improving gas barrier properties [160].

#### 4.2.2. Strategies to Improve Properties in Biobased Polymers

High research efforts have been made by the food packaging industry on modifying biopolymer properties through innovative processing techniques, nanomaterial reinforcement, antimicrobial and antioxidant incorporation, or polymer blending [161]. In this respect, various strategies for the barrier property improvements are found in the bibliography as explained below, such as melt blending, multilayer, surface coating, and nanotechnology.

##### Blending, Compounding, and Modifications

Blending and compounding are promising alternatives to enhance the technical performance of biopolymers. Blending consists of combining two or more polymers to generate a new material with specific targeted properties. In this respect, compounding includes the incorporation of additives and fillers into the reference polymer matrix.

Referring to the desired enhancement in the technical properties, excellent compatibility and intermolecular interactions between the components are key. Therefore, some cases may need proper compatibilisation (e.g., addition of graft or branched copolymers, or reactive compatibilisation) to obtain a blend with suitable performance [162].

As a result, specific combinations of additives and other biobased materials can improve certain properties. For example, the addition of plasticisers enhances processability and flexibility, whereas the addition of additives such as nanocellulose may enhance oxygen barrier performance. However, all these approaches may be detrimental on the biodegradability, which means that a balance between both sides should be considered [160].

One promising solution is starch-blended biodegradable polymers due to the compatibility between starch and other polymers such as biopolybutylene succinate (bioPBS), polylactic acid (PLA), polyhydroxyalkanoates (PHAs), and polyvinyl alcohol (PVOH). The starch content, compatibilisers, and plasticisers have a strong influence on the mechanical, thermal, and barrier properties of the blends [163].

For example, the effect of starch and different additives on the properties of PVOH-blended films was studied, showing a good compatibility and an increase in the thermal properties compared to pure PVOH. Another important advantage is that the rate of biodegradation in soil increases due to the highly hydrophilic nature of the starch, which promotes rapid bacterial diffusion and thus the rate of biodegradation. However, there is a decrease in the mechanical properties due to the intermolecular structure of starch, which is weak and highly amorphous. Then, the mechanical strength may not always meet the strict requirements of specific applications such as packaging [164].

Other limitations were identified with starch-based materials. In particular, these materials are sensitive to moisture, which can affect their performance and lifespan, especially in humid climates or in specific applications involving exposure to water such as packaging for frozen or refrigerated food items. Another shortcoming of starch is its lower heat resistance compared to conventional plastics, which limits its use in applications involving exposure to high temperatures, such as microwavable food packaging [163].

Other bioplastics that are being studied in depth are lignin-based bioplastics, which are known for the chemical interaction between lignin and the matrix. Main examples are PP-lignin or PHA-lignin polymer blends, which were found to have enhanced UV-blocking, antioxidant, antifungal, and antimicrobial properties. However, the latter all vary depending on the source of extraction of this material [165,166]. Traditionally, lignin has been obtained as a by-product of cellulose production. However, its use has grown with the development of biorefineries, which convert biobased feedstocks into useful chemicals for the plastics industry [132].

In addition, PHA-based blends are one of the most promising options due to the PHA versatility and the excellent biodegradation properties in the reference environments that they offer, as shown in Table 3. Blending PHAs with other biodegradable polymers such as polylactic acid, starch, and cellulose enhances material performance, with up to a 40% improvement in the mechanical properties [167]. Some studies have shown the improvement in the mechanical properties of PLA by blending it with PHA. In particular, some results have achieved a high tensile strength (47 MPa) by the addition of a small amount of PHB (PLA/PHB 50/10 blend) [168]. Also, a relevant improvement of PLA impact toughness (by a factor of three) and a transition from the brittle to ductile regime of fracture were found by blending it with PHB. When the mass fraction of PHB was 40, the impact toughness reached 0.40 kJ/m^2^, almost 3 times that of the neat PLA [169].

In the case of films of starch/PHA blends, they showed improved mechanical and water barrier properties compared to the pure starch. However, the compatibility between starch and PHA is poor due to the differences in polarities, since starch is hydrophilic while PHAs are hydrophobic. Furthermore, it is necessary to improve compatibility, generally by the incorporation of compatibilisers or cross-linking agents (such as citric acid, adipic acid, borax, and boric acid) to promote grafting or cross-linking reactions at the interfaces between the polymeric chains. Interestingly, the addition of cross-linking agents to the starch/PHA blends resulted in enhanced thermal stability and elasticity [170].

It was demonstrated that employing a blow moulding technique for fabricating these starch/PHA blend-based bioplastics increases the tensile force and modulus of elasticity [170]. Sun et al. synthesised the hydroxypropyl distarch phosphate (HPDSP)/PHA films by film blown extrusion. They observed that a blend containing 12% PHA exhibits high tensile strength (3.75 MPa) and light transmittance [171].

Moreover, the incorporation of antimicrobial agents and essential oils was shown to extend shelf life by 25% while keeping food safety standards. The main limitations to exploit the potential of PHAs in food packaging are the scalability of PHA production, particularly in terms of cost-effectiveness and the sourcing of sustainable carbon materials. In addition, deep studies are required to assess the real-world performance of PHA-based packaging across various applications and conditions, and to compare it with other biodegradable materials [167].

##### Physical and Chemical Modifications

Researchers have explored various physical and chemical modifications to enhance the properties of biobased plastics. Examples of these are ultrasonication and autoclaving, which were shown to improve the physicochemical properties of biobased polymers such as starch films.

In this respect, ultrasonication primarily targets the amorphous regions of the starch granules. As a result, porous surfaces are mainly produced with changes in pasting properties, solubility, or swelling capacity [172].

On the other hand, chemical modifications can act on the abundance of -OHs and their derived functionalities of some biobased polymers. As a result, hydrophilicity or water vapour barrier properties can be modified by methods including grafting, acetylation, and alkylation.

Alternatively, other studies have proven the chemical modification of biobased polymers via covalent functionalisation, introducing stable and permanent functionalities, such as cellulose and starch. For example, the esterification of cellulose has improved its hydrophobicity [173], while cross-linking starch with substances like citric acid has resulted in an improvement of water vapour barrier properties [172].

##### Incorporation of Nanoparticles

In recent years, there has been a growing interest for nano-scale particles as functional additives for biobased polymers and its related coatings. These aimed to improve barrier and UV properties, which added to the antimicrobial properties.

Nanoparticles are particles with at least one dimension smaller than 100 nm. In food packaging, they are usually selected by distinguishing between the organic and inorganic families. In the case of the organics, they can be classified according to their dimensionality as zero-dimensional (0-D), one-dimensional (1-D), two-dimensional (2-D), and three-dimensional (3-D) [174]. They can be made up of proteins, carbohydrates, lipids, polymers, or other organic compounds. In addition to their different forms, they can be applied in food packaging as nanocapsules, with some non-toxic variants but light and heat sensitive properties. On the other hand, inorganic nanoparticles include metal and metal oxide nanomaterials. These inorganic variants differ from the organic ones in their superior stability, their hydrophilicity, non-toxicity, and biocompatibility with living systems [175].

In this respect, their use can have significant results on the properties of the resulting bioplastic or coating. In the case of mechanical properties, the biopolymer–nanoparticle interaction can contribute to an improved stress transfer. Additionally, in the case of barrier properties, the presence of nanoparticles in the bioplastic matrix can result in a more complex permeation through the material. In the case of gas diffusion, the incorporation of nanoparticles with this function results in the so-called high barrier [176].

In terms of improving barrier properties, the incorporation of nanoparticles into the polymer matrix reduces the available diffusion area introducing impermeable particles and creating a so-called tortuous path for the permeants.

In the case of organoleptic properties and the preservation of perishable foods such as fish or meat, nanoparticles can play a role in reducing microbial activity. For example, the incorporation of silver, ZnO, or TiO_2_, was correlated to reduced pH changes in packaged seafood. This is due to their effect on microbial growth, resulting in a later and/or reduced formation of alkaline by-products such as ammonia (NH_3_) and trimethylamine [176].

As a result, several fillers were tested, such as zinc oxide, copper oxide, nanocellulose, lignin nanoparticles, titanium dioxide, organoclays, nano-encapsulated garlic essential oil, or chitosan nanoparticles. These have demonstrated an improvement of the water and oxygen barrier properties in biobased films. Moreover, some of these nanoparticles, such as ZnO, copper, and chitosan, also have antibacterial properties, extending the shelf life of food products, especially fruit and vegetables [177,178].

On the other hand, the incorporation of nanoparticles such as SiO_2_, Al_2_O_3_, TiO_2_, or Fe_2_O_3_ into Poli-L-lactide (PLLA) has demonstrated an improvement of the barrier properties, decreasing the water vapour permeability (up to 18%) and the wet oxygen permeability (up to 9%). Also, the incorporation of CaCO_3_ nanoparticles up to 5 wt% decreased the gas permeability to O_2_ and CO_2_ [176].

##### Multilayer Structures

As aforementioned, multilayers combine two or more materials with specific and complementary properties [155]. This results in lower needs for packaging material, with improved barrier properties [157,179] among others such as sealability. In this respect, the desired functionalities can be obtained depending on the design in the sequence of layers.

Various multilayer films were developed based on biobased polymers, combining other biodegradable polymers, such as PLA, PCL, and PVA; organic compounds, such as lauroyl arginate ethyl, carvacrol, natural plant extracts, and essential oils; and inorganic particles, such as nanoclays, silver, and metal oxides. As a result, these multilayer films improve the barrier properties against gases, water vapour, or even oil and UV light. Also, they can be enhanced with antimicrobial and antioxidant agents to increase shelf life. For example, multilayer films consisting of polylactic acid (PLA)/fish gelatine (FG)/PLA achieved an oxygen and water vapour permeability 8 and 11 times lower, respectively, compared to the reference monolayer of PLA [180]. Furthermore, bilayer films of zein and starch increased water barrier properties compared to the starch monolayer reference [181].

In this respect, a strong adhesion between the layers is crucial for the technical performance of the multilayer structure and its characteristics. The latter is difficult sometimes due to the intrinsic hydrophilicity of some biobased polymers (e.g., PHA, PLA, or natural polysaccharide-based polymers). Finally, strategies such as corona and plasma treatments are also applied to improve the adhesion between hydrophobic polymers [179].

##### Coatings

The coating technology is another promising solution to improve the barrier properties of biopolymers. In addition, it can enhance other important properties such as mechanical, e.g., tribological performance and tensile properties; optical, e.g., gloss and haze; surface, e.g., adhesion and sealability; thermal; or others such as antibacterial or antimicrobial [182].

The coating process consists of applying a layer over one or both sides of a surface, which can be a film made of plastic or other materials such as paper or paperboard. In this regard, different coating technologies can be applied such as chemical or physical vacuum deposition, solution coating, electrohydrodynamic processing, melt extrusion, or hot pressing.

On the other hand, the coating materials are also important. This is the case of metallised or inorganic nanosheet coatings, which are the only current viable solutions achieving food packaging requirements on bioplastic films.

Alternatively, polymeric barrier coatings such as polyfluoroalkyl (PFA) substances, coated in paper, films, or the epoxy inner coatings of cans, have demonstrated to affect health and their recyclability [183]. On the other side, polyvinyl alcohol (PVA) is a more sustainable and widely used biodegradable coating for flexible/rigid paper and plastic packaging to decrease oxygen permeation, despite its fossil origin.

Nevertheless, the trend in the industrial and academic sectors is the transition from fossil-based coatings to biobased and even biodegradable alternatives. In this respect, there are several challenges to overcome such as tunning the desired biodegradability, the large-scale production, the heat-sealability during service time, or the migration of the coating into the packaged food [184]. Regarding the materials used, biobased coatings can be made from several biopolymers, including, biobased polyesters or polymers from nature.

##### Biobased Polyester-Based Coatings

Biobased polyesters or multilayer structures combining options such as PHAs, PLA, or bioPBS coated on conventional polymer substrates were shown to improve barrier properties against water vapour and/or oxygen.

Polyhydroxyalkanoate (PHA) coatings provide good barrier properties against water vapour because of their intrinsic hydrophobicity. For this reason, PHA-coated paper is being investigated as an alternative to other fossil-based coatings.

In parallel, Polylactide (PLA) and PLA-blended coatings are also being investigated since PLA is one of the most mature biobased polymers. Some methods to improve PLA barrier properties including dispersing an additional phase (such as fibres, filler, additives, or biopolymers) into the PLA matrix to produce bio/nanocomposite blends, on the one hand, and modifying the PLA surface (e.g., by wet casting, hot pressing, electrospinning, or co-extrusion), on the other [184]. In fact, polylactic acid already has commercialised examples such as coating for paper packaging, stand-up bags for dried fruits, or paper glasses, and provides a high water vapour barrier but low oxygen barrier [185]. Also, starch-based and polyhydroxyalkanoate aqueous coatings were demonstrated to provide good grease resistance and odour protection, but do not achieve water barrier properties [66].

##### Coatings from Nature

Polysaccharide-based coatings, with their main examples being cellulose, starch, chitosan, alginate, and pectin, provide an effective barrier against gases due to their hydrogen-bonding packing network structure. However, limitations include their low moisture barrier capacity derived from their hydrophilic nature or their brittleness. For this reason, their applications are generally addressed by the combination of polysaccharides with other biopolymers.

On the other hand, lipid-based coatings provide excellent water barrier properties due to their hydrophobic characteristics, which give them a great potential for packaging applications. Lipids can be extracted from diverse biomass such as animals, insects, or plants, with their most common examples used in food packaging being vegetable oils, natural waxes, cutin, and essential oils [184]. In the market, Solenis Topscreen biowax can be found containing by up to 100 percent biobased content derived from vegetable oils. It is used in paper and paperboard coatings, with the added value of being recyclable and compostable [186].

Alternatively, protein-based coatings can be derived from plants and from animals, in the forms of wheat gluten, soy proteins, corn, or zein. These kinds of protein-based biopolymers show high barrier properties against oxygen. For example, soy protein-based films show a reduction of 540, 500, and 670 times of oxygen permeability in comparison with starch, polyethylene, and pectin, respectively [184].

However, the main challenge of these protein coatings is their brittleness and poor water barrier properties due to their intrinsic hydrophilicity. For this reason, several studies have focused on different strategies to overcome this challenge such as the chemical modification of proteins and the addition of plasticisers or another polymer. For example, the coating of plasticised whey protein on a commercial PLA film improved oxygen barrier properties by up to 84% [184].

Protein coatings such as the zein-based coating, such as FloZein commercial products, were applied on cardboard containers for greasy food and as a moisture and microbial barrier for fresh and dried vegetables [187].

## 5. Production Techniques

### 5.1. Classical Manufacturing Routes

**Figure 5:**
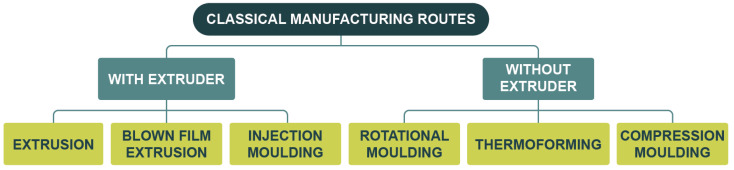
Classical manufacturing routes with or without extruder.

Food packaging still relies on classical plastic processing methods such as extrusion, injection moulding, or thermoforming. All of these are generally geared toward high volume production and the use of thermoplastics supplied in the form of pellets, powders, or granules. In this respect, classical manufacturing routes can be divided into two main categories: extruder-based and non-extruder based. Those using extruders are extrusion, blown film extrusion, and injection moulding. While the non-extruder-based processes are thermoforming, rotational moulding, and compression moulding, as shown in Figure 5.

#### 5.1.1. Extrusion

**Figure 6:**
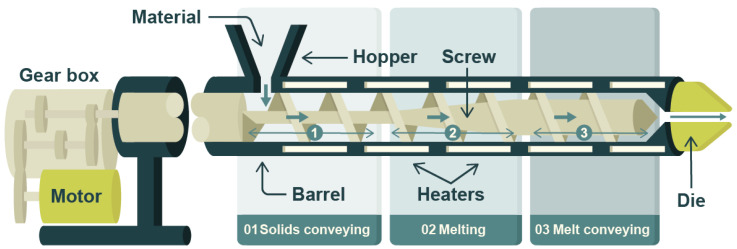
The main stages affecting the functioning of an extruder, adapted from [188].

It is a high volume manufacturing technique in which a thermoplastic polymer is homogeneously mixed by the shear forces of a screw. The molten material is then pressed against a die or forming head, from which it is ejected in the desired shape. This type of machine is divided into single-screw, twin-screw, and multiple-screw versions. All of these consist of only three main stages during its functioning, which are solids conveying or metering (1), melting (2), and melt conveying (3), as shown in Figure 6. As such, they constitute the main bone of contention in the extrusion processing of polymers. In fact, despite the significant improvements over recent decades, they still pose challenges in terms of thermal monitoring and controlling.

In this respect, the main challenges that operators may face are melt flow, thermal stability, homogeneity, or the consistency of the flow rate [188]. The latter depends mainly on parameters such as screw geometry, temperature and pressure, and the resulting properties of the extruded material.

Therefore, theories of optimisation methods, based on the mathematical description of the process, have emerged. These solve real extrusion problems addressed not only for single-screw extruders, but also for twin-screw extruders, their dies, and calibrators. In fact, they have proven to be a more efficient solution than the application of empirical knowledge, the use of simulation tools, or trial and error.

These theories can be inspired by the proposal of Tadmor and Gogos [188], which stated the decomposition of polymer processing into its elementary steps for a quantitative analysis. The latter combines the principles of transport phenomena, fluid mechanics, heat and mass transfer, polymer melt rheology, solid mechanics, and the physics and chemistry of polymer mixing.

Based on this, several optimisation methods were developed to define the best Pareto solution for improving the extrusion process. These generally involve multiple and sometimes contradictory criteria, e.g., trying to maximise yield, while minimising viscous dissipation and mechanical energy rates. The latter is known as multi-objective optimisation.

Therefore, a statistical analysis approach can be used to find the best solution. The latter is usually based on experimental data and computational results that can be used to derive a mathematical model that correlates the objective function with the decision parameters. Alternatively, a method can use basic information to perform a guided search through an algorithm. The latter provides random iterations that are later guided based on local information until the final decision [189].

Twin-screw extrusion differs from the single-screw extrusion in that it provides a more homogeneous mixture and faster achievement of melt conditions. A distinction is made between co-rotating and counterrotating versions, depending on the relative direction of rotation between the screws. The screws can also be intermeshing or not. As a result, this type of equipment is most commonly used for thermally sensitive polymers and compounding operations involving the addition of fillers.

To model extrusion processes, several events need to be described, such as solid polymer transport or plasticisation on melt flow. In addition, twin-screw extrusion has different flow dynamics than single-screw extrusion due to the additional screw and the resulting different configuration. This leads to a more complex mathematical description of the flow inside the barrel, and, consequently, to a less developed theory.

In this respect, recent innovations have focused on the prediction of the polymer behaviour in the extruder, and the design of new screws [190].

Computational fluid dynamics (CFD) has proven to be a powerful tool for the modelling of the extrusion process. It helps to study the process in detail by calculating, e.g., the distribution of shear stress, shear rate, viscosity, or residence time. Also, 3D finite element method (FEM) calculations can also be used to describe other phenomena such as velocity and temperature distributions or pressure/flow rate ratios. However, a thorough understanding of the procedures to be followed, the boundary conditions, and the correct interpretation of the results is required [191].

In the field of twin-screw extruder screw design, hyperbolic channels have been a breakthrough. This innovative design achieves a significant improvement in dispersive flow compared to the conventional kneading block. Therefore, hyperbolic provides a better dispersion, but with more energetically inefficient shear flows for dispersive mixing [190].

#### 5.1.2. Blown Film Extrusion

Blown film extrusion is the main processing technique used in film production. It can produce a wide range of products, from monolayer, e.g., for plastic bags, to multilayer, e.g., for smart packaging. There are commercial lines that can co-extrude up to 11 layers.

Its function is therefore highly dependent on the extrusion die, which uses a combination of circular shape and internal air pressure to create a bubble-like film expansion during the extrusion. The film with the desired dimensions and layers composition is then cooled and folded.

The main developments in this technique can be divided into two main areas: the use of air-guided systems and the optimisation of the die design through computer simulation.

**Figure 7:**
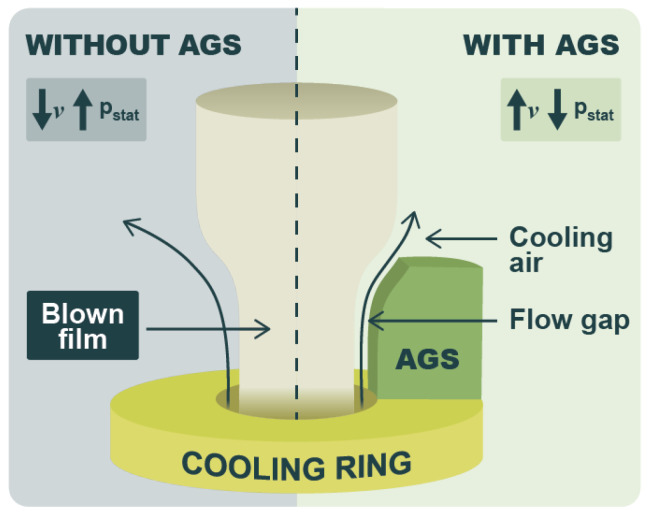
Air guiding system (AGS) to enable higher heat removal out of the film bubble by the venturi effect, adapted from [192].

In this respect, the University of Aachen in Germany, has developed an improvement consisting of a flexible air duct [192], as shown in Figure 7. This is able to enclose the film bubble extrudate in its expansion zone creating an air flow space between the membrane and the bubble due to the Venturi effect. The result is a better cooling effect and an increase in productivity by up to 62%. However, the movement of the air at high speed affects the thickness uniformity.

Alternatively, Ansys Fluent–Fluid Simulation Software 2024 R2 was also used for co-extrusion, allowing a rheological study of the blown film in order to optimise the transition between the different sections [193].

Finally, there is also the situation that the industry is facing with stricter regulations and more environmentally friendly demands from consumers [10]. As a result, new technologies are being considered for film production, with the latter due to the more sensitive nature of bioplastics or the introduction of recycled. This leads to the consideration of other technologies with applications in both film production and coatings, such as non-solvent phase inversion (NIPS) or thermally induced phase inversion (TIPS) [194]. However, they still require further development in terms of the surface roughness produced and their barrier properties [195].

#### 5.1.3. Injection Moulding

Injection moulding is one of the most common plastics processing techniques worldwide. This process combines an extruder with a moulding stage. As a result, its choice combines affordability and lightness for parts with low mechanical requirements, such as food packaging. This technology can be considered consolidated. However, challenges such as waste generation, material use, cooling cycle time, or energy efficiency still leave room for optimisation [196]. Some examples of these are summarised as follows:

- **Microcellular injection moulding (MIM)**. This technology works during mould filling by injecting a dissolved gas into pressurised molten polymer, which is quickly depressurised [197]. This allows the mould cavity and material distribution to be controlled. This results in a material reduction of between 30 and 40% and a higher impact strength. The latter due to an internal structure in the moulded parts consisting of a high density of micro-voids [198].
- **Rapid Thermal Cycling Moulding (RTCM)**. This technology involves the rapid heating and cooling of the mould to improve part quality and the efficiency of the moulding process. It consists of the heating of the material to its forming temperature until the mould is filled, and then rapidly cooling it. As a result, the moulded parts achieve very good aspect quality, with high gloss and no defects such as joint lines or collapses [199].
- **Multicomponent Injection Moulding**. This consists of combining different materials that are injected simultaneously during the moulding process. As a result, moulded parts can have different colours, chemical properties, hardness, textures, or other characteristics [200]. However, this technology still faces challenges such as setting the processing parameters of simultaneous components and equipping it with multiple injection units.
- **Reaction Injection Moulding (RIM)**. This technology produces plastic parts by a reactive in situ polymerisation. It therefore uses two or more liquid monomers, which must be carefully well mixed, as its main technical barrier.

On the other hand, injection moulding is associated with a high environmental impact. Not only does it generate waste, but it also requires a large amount of electricity [201].

Injection moulding involves the use of large quantities of raw materials, contributing to further material depletion. In addition, the use of a wide range of chemicals to achieve the desired properties, together with the resulting waste by-products, is an environmental hazard.

In this context, more sustainable manufacturing practises, such as the use of renewable materials, energy management, process optimisation or the introduction of recycled waste, should be considered.

#### 5.1.4. Thermoforming

Thermoforming is a technology that involves forming a heated plastic sheet into a desired container shape. The thermoformed sheet is then transported to the trimming station where individual containers are created, such as food packaging. As a result, this technology offers mass production of parts with excellent barrier properties for food preservation.

On the other hand, the technology faces challenges such as thickness uniformity [202], which is largely influenced by the processing parameters. In this respect, simulation techniques were introduced to address this issue. For example, a multi-objective optimisation strategy based on evolutionary algorithms was used to determine the final thickness distribution of the part along the surface, with the aim of demonstrating the validity of the proposed methodology. The results of this analysis can be used for the production of parts with the least amount of material, while ensuring the appropriate characteristics of the final part. In fact, it is possible to obtain a reduction of around 30% in the volume of the material [203].

In addition, the use of recycled blends with virgin material results in unstable temperature and flow rates, which is due to the material variations. Therefore, the use of intelligence databases, combined with DSC and TGA analysis, should be considered to assess the validity of the sample [202].

The use of bioplastics was also considered, and industries are making efforts to migrate their processes from fossil-based to bioplastics. In this respect, polylactic acid stands out as the most promising among other bioplastic alternatives. This is due to its compostability, in addition to its good stiffness and transparency [204].

#### 5.1.5. Rotational Moulding

Rotational moulding is a unique low-shear process used to produce hollow parts. It consists of melting a polymer, usually in powder form, which takes the shape of a rotating mould. The mould is heated to the melting temperature of the polymer and then cooled for the demoulding process. In this respect, it is an excellent method for batch processing, minimal waste generation, and stress-free parts. However, the process has drawbacks such as long cycle times and a limited range of materials compared to other processing methods [205].

However, there is a lack of understanding on the behaviour of the phase transition for the rotomoulding procedure. In this respect, temperature variations in both the heating and cooling phases impact the final product quality [206]. To gain in depth knowledge in this area, research has focused on the evolution of external and internal temperature profiles during the cycle time, based on the geometry of the mould. Therefore, geometry optimisation led to a reduction in the cycle time between 18% and 28%.

Apart from these, there are additional studies on the accurate prediction of temperature using computational fluid dynamic (CFD) software. The latter resulting in near-wall treatments of the duct, and the correlation between insulation thickness and the heat sources. As a result, a methodology for the optimum insulation thickness (OIT) was established with improvements in the reduction in the associated greenhouse gas emissions [206].

### 5.2. New Manufacturing Routes

Apart from the consolidated classical manufacturing routes, existing and other innovative technologies that have emerged recently may be applied to food packaging in the near future at an industrial scale, such as electrospinning, additive manufacturing, plasma, microwave heating system, or ultrasound. Their state of the art is discussed below.

- **Electrospinning (ES)**. It is a promising technology that can be used either to produce membranes or to coat surfaces, such as food packaging films, with tailored properties. This process was developed in the early 19th century, although its industrial application to food packaging is still in its infancy [207]. It consists of the production and deposition of nanofibres onto a surface. The process works by applying an electrical potential between a syringe and the surface of the material. A solution is released in the form of a tunable Taylor cone. From this, extra-fine threads of fibre are projected across the surface by a mixture of forces such as electrostatic, drag, and gravity. The fibres can be produced in a variety of forms, using the three main categories of ES [208]: uniaxial, coaxial, and emulsion ES. Uniaxial ES is the most widely used technique, in which a homogeneous solution of active material is applied with a single needle. Coaxial ES uses an additional coaxial needle to produce nanofibres with a “core-shell” structure. Emulsion ES uses a pre-prepared water-in-oil or oil-in-water emulsion that is mixed in a single needle.

Therefore, the application of these categories of ES to food packaging offers a promising solution for the development of active and smart surfaces for food packaging. Tailored biopolymer nanofibres with antimicrobial, antioxidant, high temperature, and high humidity resistant properties can be developed. However, there are barriers to their implementation in food packaging on an industrial scale. A key point is the need for material development, especially in the case of hydrophobic electrospun nanofibres and their stabilisation to avoid the risk of these nanofibres migrating from the packaging to the food [209], or the development of eco-friendly solvents to avoid toxic organic solvents. There is also a need for process optimisation, such as achieving uniformity and consistency in the projected nanofibre properties. In addition, nozzle clogging is another issue that needs to be addressed in order to scale up the use of nanofibers for the production of food packaging on an industrial scale [210].

- **Additive manufacturing (AM)**. This is an emerging technology that consists of the controlled deposition of materials to produce parts. This technology is characterised by its ability to produce complex geometries, to reduce material waste, or to restore damaged parts [211]. However, it still needs to be further developed in the following areas, which are in fact several fields of research: the incorporation of a greater number of materials, especially for high strength applications; improving the existing anisotropy, present in the Z axis, by modifying the process; or the introduction of multi-nozzle print heads for the injection of the reinforcement and the polymer matrix. On the other hand, in order to meet high-end applications, AM should improve its quality by eliminating internal stresses, improving surface finish, and achieving reliable thin-walled structures. This may also include improving post-processing heat treatments or removing support materials.

It is worth noting that three of the seven types of AM are laser-based, such as directed energy deposition, powder bed fusion, and vat polymerisation. These have laser powers ranging from 1 W to 6 kW in wavelengths ranging from ultraviolet (354.7 nm) to infrared (10.6 nm). In this respect, lasers have as much potential as AM itself. Their improvement could address current challenges such as energy efficiency, process repeatability, layer/deposition rate, or high energy consumption, as well as additional functionalities such as cutting or drilling [212].

- **Plasma**. Since its invention in the 1950s, plasma has undergone considerable development and is used in many fields, including electronics, microelectronics, and medicine [213]. It consists of the ionisation of gas molecules with a controlled charge of energetic electrons and positively and negatively charged ions [214]. It can be used to tailor different treatments, modifying materials with a selective flux of energy and matter. This has led to innovations in several fields such as thin-film production, fibre processing and, more recently, improved polymer wettability.

A distinction is made between thermal and cold plasmas, with the first having a higher energy density during the treatment. In the case of cold plasma, with its variants of physical vapour deposition (PVD) and chemical vapour deposition (CVD), it is a promising technology with current applications in the production of polymer coatings [213].

- **Microwave heating systems**. It is a growing trend in polymers, with implications for both the polymer chemistry and its processing. This is due to its design as a heating method with reduced heating times and a lower carbon footprint than other conventional methods.

It is easily applicable to polar polymers for treatments, such as drying, polymerisation, functionalisation, or chemical modification. In the case of non-polar polymers, which do not interact directly with microwaves, it can also be applied by introducing microwave absorbers within the polymer structure. However, this technology faces challenges in controlling and regulating temperature during the process. In this sense, modelling offers a powerful approach that could help to understand the polymer behaviour under microwave irradiation [215].

- **Ultrasounds**. This technology has experienced considerable growth in recent years because it is capable of producing both chemical and mechanical effects in liquid media. As a result, it can be used for chemical reactions, polymer synthesis, nanoparticle synthesis, colloids, food processing, etc.

In fact, the recent use of high frequency ultrasound (>100 kHz) for polymer synthesis has initiated a new trend, especially in chain growth polymerisation and reversible deactivation radical polymerisation (RDRP) [216]. It also finds applications in material property modulation, directed assembly, sensing, and biochemical process activation, thus also developing smart properties for polymeric materials.

On the other hand, ultrasonic processing is a flexible green technology for energy efficient processes. However, its main challenge is to be scaled up to industrial systems and further development is needed [217].

- **Surface chemical modification by grafting of active compounds.** In addition to other advanced surface treatments in film packaging, such as corona or plasma treatments, their chemical modification by chemical grafting may grow in the coming years due to the expected market growth of polysaccharide-based bioplastics (such as starch, cellulose, chitosan) and the popularity of active packaging. The grafting of compounds occurs due to the inherent chemical structure of such bioplastics with their inherent functional groups that can be converted into active sites for modification. This can be conducted by various methods, such as enzymatical, physical, and chemical. One of the most extensively researched is the chemical free radical grafting, which can significantly improve the mechanical, barrier, and antimicrobial properties of these films, extending their applicability to the food and pharmaceutical industries. The latter may find a place in the food packaging industry due to the recent development of active films with the pretended incorporation of biobased additives with antioxidant and antimicrobial properties. These properties are achieved through the action of polyphenols, which are sensitive to temperature and the related heat transfer that occurs during melt processing, e.g., extrusion. However, the mechanisms behind the grafting reactions and their effects on the stability of the intended polyphenol-based compounds need to be further researched [218].

## 6. Future Trends in the Development of the Food Packaging Industry

### 6.1. Smart and Intelligent Packaging

Traditional food packaging protects its contents passively by providing a barrier against external influences. Intelligent packaging refers to packaging which monitors “the condition of packaged food or the environment surrounding the food” [219]. It provides information but does not interact with the product. Intelligent packaging working synergistically with an active component, i.e., a packaging component that actively responds to external conditions and interacts with the product, and is referred to as smart packaging [220]. Smart and intelligent packaging has seen an introduction in Asian and North American markets; a widespread adoption in the European market is yet to be seen [221].

Intelligent packaging can be realised as sensors or indicators, which can monitor the state of the food itself (e.g., pH) or the conditions in the headspace (atmosphere, temperature, volatile substances), or as carriers of data in form of barcodes or tags [220,222]. Sensors respond with a quantifiable signal proportional to a measured quantity (biological, chemical, or physical). Indicators respond to either the presence or absence or to the concentration of a given substance, typically via changes in colour or intensity. Thus, they are more immediate than sensors and more accessible to consumers.

Temperature control and the time–temperature profile a food product is exposed to are critical to the preservation of freshness and the extension of shelf life, especially for chilled and frozen foods. Thus, time–temperature indicators (TTIs) are a valuable application of intelligent packaging. TTIs frequently contain components that undergo chemical or physical changes depending on the time–temperature profile, leading to a visible change, thus providing a clear indication for potentially harmful temperature exposure [223,224].

The deterioration of food is often accompanied by the production of certain compounds or changes to the pH value. Freshness indicators respond to either specific molecules or the pH value of the food, usually by colour changes, thus providing direct information on the freshness of the packaged food [225,226].

Similarly, gas indicators and sensors respond to the presence of certain gases, most commonly oxygen or carbon dioxide. Their applications lie usually in conjunction with MAP or active packaging like scavengers [223,226].

For sensors, different operating principles exist, most importantly chemical, electrochemical, and optical [220]. Chemical gas sensors can also respond to the presence of volatile organic compounds, thus linking directly to the freshness of the product. However, chemical sensors tend to be too large and costly for most packaging applications. Electrochemical sensors consist of electrodes, with the working electrode responding to the measured quantity. Electrochemical sensors can be built from a range of materials; for the food packaging sector, carbon nanomaterials are a promising candidate, as they are lightweight, sensitive, and robust [227]. However, for their widespread use to be feasible, further development is necessary. Optical sensors react to the presence of their specific target by producing an optical signal, e.g., colour, luminescence, or fluorescence, or by changing their optical properties. The signal can be measured by means of a photodetector or, in some cases, seen by the naked eye. A relatively established application is the use of fluorescent oxygen sensors. Other emergent optical sensor technologies are used for the detection of volatile organic compounds [228].

Data carriers, like RFID (radio frequency identification) or NFC (near-field communication) tags and barcodes, including QR, do not provide real-time information on the packaged good, but rather allow for traceability of the package. These technologies are applied mainly in transport packages, not so much in primary packaging, and can be combined with sensing capabilities to allow for contactless readout and control [220]. RFID tags can be used at a retail level for convenient checkout, but this application is not yet common in the food sector [229].

Given the extra expense in terms of resources required to produce smart and intelligent packaging, reusable packaging provides a compelling application for these technologies compared to disposable packaging. Hakola et al. performed a case study on the application of smart tags on reusable packaging, specifically considering the durability of the tags under the handling and washing steps expected for reusable packaging [230]. The study included laser-etched QR codes printed on NFC antennae and affixed to RFID tags. All tested technologies proved to be resistant to damage during handling, including microwaving and cleaning. The QR codes provide the least amount of readable information, but do not impact the recyclability at the end-of-life of the reusable packaging unit. NFC antennae and RFID tags can hold more information but may impact recycling unless they are removable.

While the sensing and tracing capabilities of intelligent packaging have the potential to curb food waste and increase food safety, thus saving resources and costs, the adoption of intelligent packaging incurs additional expenses. As Chen et al. point out, printable electronics provide a cheap, scalable and—with over a decade of development—relatively mature option for the implementation of intelligent packaging in the food value chain [231]. The field of printable electronics remains in a state of highly active and dynamic development [232]. However, the complexity of food supply necessitates collaboration between the multitude of actors involved and a holistic approach [221].

### 6.2. Digitalisation of Packaging Production

Industry 4.0 was first introduced by the German Federal Government and can be referred to in different ways, such as the fourth industrial revolution, smart factory, intelligent factory, Internet of Things (IoT), or cyber–physical system (CPS). This new concept of industry is based on the combination of the connected data, analytics, human–machine interaction, and the use of advanced engineering. As a result, its introduction is expected to bring economic opportunities and address social and environmental issues by reshaping industries and integrating information systems into their activities [233].

From an economic point of view, several opportunities were identified, such as the creation of competitiveness, due to a greater flexibility, production efficiency, and reduction in time and logistics costs. In the case of the environment, it is expected to reduce greenhouse gas emissions and waste generation [234].

Food packaging, as another industrial sector, will contribute significantly to these challenges. In the context of Industry 4.0, the aim is to improve the efficiency of production processes and enhance food safety, its quality, and traceability. This can be achieved by combining several advanced technologies, such as nanobiotechnology, Internet of Things (IoT), artificial intelligence (AI), big data, smart sensors, blockchain, or 3D printing [235]. In the case of IoT and smart sensors, the monitoring process of food storage will be more efficient and food loss and waste will be reduced. In addition, AI will help predict consumption trends and optimise the amount of food and its packaging to be produced. On the other hand, blockchain offers the possibility of efficient traceability of packaging. Meanwhile, nanobiotechnology can be used to turn food waste into upcycled materials and nanosensors. However, Industry 4.0 technologies for the food packaging sector are still under development, with the main barrier being the investments required.

The next step is Industry 5.0, which aims to combine human creativity with intelligent machines in order to achieve efficient and sustainable improvements over 4.0.

#### 6.2.1. Digital Twins

Digital twins (DTs) are a powerful technology that is widely used in various industries, particularly manufacturing. They can monitor performance, optimise processes, simulate results, or predict potential failures. In addition, their applicability extends to the entire product life cycle, from design, manufacturing, delivery, use, and end-of-life [236].

Examples of its application in the plastics processing industry are the rapid start-up of manufacturing processes, such as a case study in PVC tube extrusion [237], or the design and testing of a digital twin to assess the quality of an extrusion process [238].

In the case of processing technologies such as plastics compounding or injection moulding, digital twins can play an important role in solving physicochemically complex problems that are difficult to optimise. However, their success depends on the availability of large databases. To overcome this problem, a hybrid solution was proposed that combines real experiments with the modelling of the physicochemical process and subsequent generation of a data cloud [239].

Moving on to injection moulding, the digital twin can be applied in the forms ranging from mould design, through mould making to the injection moulding process. In this respect, IoT and CPS can play an important role. However, its realisation still needs to address challenges, such as manual data acquisition, the implementation of sensors in the injection moulding machine, the development of real-time automated visual inspection systems, or the optimisation and prediction analysis model [240].

#### 6.2.2. Machine-Aided Learning

Machine-aided learning (ML) can be defined as a field of study in which algorithms are used to learn data without being explicitly programmed. This results in the possibility of solving problems without the need for detailed domain-specific knowledge [239]. In this respect, the application of ML tools can be divided into a set of steps, such as data collection and analysis, model training, model testing, and model deployment. As a result, the model will be able to make predictions.

The integration of ML into the plastics industry is crucial to address the environmental concerns associated with synthetic plastics. The transition to sustainable polymers from recycled, renewable, and residual carbon resources that can be recycled, biodegraded, composted, or disposed of in an environmentally sound manner is a promising solution to reduce plastic pollution. ML accelerates the development of sustainable polymers, reducing time and costs throughout their entire life cycle, from design and formulation to processing, application, and end-of-life management.

#### 6.2.3. Decision Support Systems

A decision support system (DSS) is an information system capable of assisting the user in choosing the optimal solution, in a shorter time. In this respect, a good DSS should be based on a system analysis approach, capable of acquiring, representing and analysing knowledge, flexible and able to deal with missing or uncertain data, user-friendly, and proficient at producing a useful output. Several initiatives have been carried out in the plastics sector, such as material selection, recycling plants, and waste management [30].

## 7. Main Technical, Economic, and Regulatory Challenges Facing Adoption of New Packaging Designs and Materials

The current European food packaging industry is completely overtaken by commodity oil-based plastics such as PE, PP, PET, and PVC, with over 40% of the global market share. They offer exceptional barrier properties, mechanical properties, and geometric and thermal stability, added to an affordable cost. However, sustainable concerns have been imposing during the last decade and environmentally conscious regulations and consumers demand are pushing toward a migration to bioplastics. The latter offer a reduced carbon footprint added to the ability of accelerated degradation when exposed to the environment. Nevertheless, their full adoption still poses challenges such as those summarised below [103].

- **High costs, production yields, and biobased feedstocks.** There is still room for improvement in the production of bioplastics compared to oil-based plastics. Most bioplastics in the market have a higher cost compared to conventional fossil-based plastics, generally about two times more expensive than fossil-based plastics [103,241]. This is a major barrier to their adoption in the industry. In addition, their production still needs to be developed. In fact, the energy efficiency of conventional plastic production processes is higher than that of bioplastics. Alternatively, the use of biobased feedstocks must be carefully considered in order not to interfere into animal and human food production.
- **Costs of change management**. Substantial changes in packaging design, such as the exchange of established materials through new ones, often requires corresponding changes to the affected production lines. The associated costs provide economic barriers to companies and may negatively affect decisions for change.
- **Lower technical performance**. It is a general consideration that most bioplastics have lower technical properties compared to their conventional counterparts. This is the case for thermal stability, mechanical strength, heat sealability, high water vapour, and oxygen permeability. As a result, strategies such as multilayer structures have to be used, resulting in an increase in cost [242]. Reduced gas barriers may result in a reduction in the shelf life of packaged foods, thus potentially increasing food waste. On the other hand, biopolymers usually have limited applicability without modification. The quality of biobased materials depends heavily on their feedstocks, which may vary seasonally and regionally. Especially for materials with a lower maturity, the control of these factors may not be well understood.
- **Challenging processability**. Common biopolymers exhibit thermal sensitivity, brittleness, and tackiness. Their own processability is therefore challenging, and that is due to the fact that they are usually blended with others, making the process more complex. This may also necessitate the adoption of new processing equipment, possibly incurring additional costs.
- **Biodegradation and waste management**. Apart from the excellent biodegradation properties for which some biodegradable bioplastics are well known, there is a lack of consideration of the different biodegradation environments. In this respect, biopolymers may show better biodegradability in certain environments, e.g., PLA, which is biodegradable under industrial composting conditions. For this reason, another challenge could be the correct labelling of products made from bioplastics, taking into account their preferred end-of-life destination. In fact, when considering the introduction of bioplastics in the food packaging market, appropriate waste management systems should be established to take advantage of this important property [161]. Finally, there are concerns about the additives used in the processing of bioplastics. This is important because when these bioplastic products biodegrade, potentially hazardous by-products may be released. Therefore, a careful life cycle assessment should be carried out for these types of materials.
- **Reusable packaging**. In order for reusable packaging to become more widespread, it is necessary not only to design packaging that is suitable for repeated use and can withstand additional wear and tear caused by repeated use, but also to establish the necessary collection points, logistics network, and cleaning infrastructure required (depending on the exact implementation of reuse). In addition, the involvement of consumers is crucial to the transition from single-use to reusable packaging.
- **Regulatory constraints**. Regulations such as the PPWD on an EU level constrain design and material choices. Stipulated recycling rates require more packaging to be recycled, thus necessitating the appropriate choice of materials. Reductions in the amount of packaging proscribed by regulations generally encourage more minimalist design, while specific types of single-use containers (e.g., for fresh produce unless required by the food) face a ban entirely, encouraging the adoption of packaging-free sale or the implementation of reuseable packaging.
- **Smart and intelligent packaging**. The use of active and intelligent packaging components can face barriers from several directions depending on the specific technology. Regulations may hinder the use of sensing technology if the safety of the contact is not fully established. Intelligent packaging components affecting the traceability of individual packaging units may be affected by data security concerns and corresponding regulations. Increasing the complexity of packaging through these approaches also increases cost per unit and may, depending on the technology, affecting its sustainability.
- **Consumer response**. Changes in packaging design that affect the appearance or handling of packaging may be rejected by parts of the consumer base if not accompanied by an information campaign or clear labelling. Since consumers value the safety of the food they buy, design changes related to the material choice must be well communicated. The observed gap between consumer perception of sustainability and objective measures like LCA can also impact the establishment of new packaging designs.
- **Plastics processing.** Plastics are characterised by their ease of processing and low energy consumption compared to other materials such as steel or glass. In this respect, the plastics processing industry is currently so dependent on the classical plastics manufacturing routes, e.g., extrusion, thermoforming, injection moulding, or blown film extrusion.
  These abovementioned technologies need to be adapted to these new sustainable materials. As in the case of conventional plastics, several production problems can occur during extrusion or thermoforming, which could increase due to their sensitivity or the blending complexity of bioplastics. In this respect, several optimisation methods have been developed over the last decades that should be considered for the introduction of these materials, such as CFD or 3D FEM. With these, several computational simulations could be carried out to evaluate processing parameters, screw design, or die design, especially for blown film co-extrusion. On the other hand, injection moulding has seen the development of innovative processing methods such as the abovementioned MIM, RTCM, multi-injection moulding, or RIM. With these, advances in material efficiency and part strength, part finish, smart material part production, or alternative moulding processes, respectively, could be applied to the new requirements of bioplastics moulding for food packaging. There is also the issue of developing and introducing new manufacturing processes, such as the abovementioned ES, AM, plasma, microwave, or ultrasound. In this respect, ES still needs further material development and process optimisation for its industrial scalability, and AM still has room for improvement, with further development of its lasers and manufacturing routes. This could lead to promising results such as waste reduction, the production of complex geometries, or the restoration of parts. In the case of plasma, it offers a wide range of possibilities, especially in the case of thin-film production, surface treatment, or coating production. Meanwhile, microwaves and ultrasound could offer energy efficient ways of producing materials and modifying their properties. The choice of materials, the required application of manufacturing technologies, consumer interaction, regulations, and sustainability concerns all need to be addressed in the packaging design, with the cost of the packaging being a constraint. Design trends point to improving sustainability by reducing packaging complexity, facilitating recycling, and selecting materials for both sustainability and functionality. The use of monomaterials wherever possible, with thin barrier layers where necessary, is a continuing trend in packaging design.

## 8. Potential Future Developments in Food Packaging

The food packaging industry needs to move away from oil-based plastics to more sustainable alternatives. As discussed above, there are several challenges to overcome in order to make this a reality. This is where Industry 4.0 comes in, offering the opportunity to empower the industry by connecting it to real-time data and analytics, in addition to the implementation of advanced technologies. As a result, its implementation will lead to further advances in greenhouse gas emissions or waste generation. With the use of IoT or AI, the food packaging industry will be able to adapt food packaging to smart functions and could also monitor demand trends to optimise resources according to the demand.

On the other hand, DT, ML, and DSS could play a key role in optimising plastics processing to these new material requirements, for example, solving issues during production or reducing time losses.

The integration of QR codes and printed electronics can aid in the implementation of Industry 4.0 without compromising recyclability or biodegradability. QR codes can be laser-etched or printed on using suitable inks, while printed electronics can use materials like carbon nanoparticles that do not interfere with end-of-life options. Thus, these technologies do not hamper the trend toward simplification in design and the use of mono-materials.

The push toward mono-materials and the use of structural design elements to reduce the amount of material needed without compromising the functionality of packaging may well expand to so far non-optimised packaging designs but will likely reach a natural limit where further optimisation without material changes will not be possible. Barrier materials, which can be applied in thin layers (e.g., lacquers), have the potential to provide sustainability-friendly solutions in this area.

As far as packaging ergonomics and inclusivity toward people with mobility issues are concerned, developments of more ergonomic approaches to packaging design may well spread to packaging solutions, which so far pose difficulties. Considering that many societies are increasingly skewing toward higher age groups, this is more likely.

## 9. Predictions About Emerging Innovations

Due to their wide applicability, functionality, and low cost, fossil-based plastics still play a dominant role in the food packaging sector. With further research and development, wider adoption, and the optimisation of production, it can be expected that biobased polymers will gain increasing traction in the future. The utilisation of waste streams may open new feedstocks and enable the reduction in production costs.

A major downside of many biobased materials is their low barrier compared to fossil-based polymers, especially for water vapour. Two developments have the potential to address this issue: multilayer biomaterials and the development and maturation of new materials. If biodegradability is the goal, the combination of individually biodegradable materials to a multilayer material is likely to be biodegradable by itself. Thus, the properties of the individual components can be synergistically combined without losing biodegradability, thus making use of the benefit of multilayer materials while avoiding a major downside. Simultaneously, the development of biobased materials with good barrier properties or the maturation of the production process for known materials has a high potential to open new pathways to sustainable packaging. PEF is an example of a material with good barrier properties that can be biobased and is highly recyclable, where large-scale production is not currently available. The development of sufficient and economic production could take this material forward and have a major impact on the market.

The adoption of digital watermarks for improved sorting and developments in recycling technologies (e.g., chemical- or solvent-based recycling) have the potential to enhance the technical and actual recyclability of commonly used materials. Depending on the scalability and efficiency of these procedures, developments in these areas could enhance the sustainability of already well-established materials or open additional materials for recycling and thus a circular economy.

## 10. Conclusions

Packaging design determines the functionality and sustainability of food packaging as well as consumer interactions with it. For the latter, ease of use and clear communication of the end-of-life are key. Under the environmental impact of many types of conventional food packaging, there is a push toward simple, monomaterial packaging for enhanced recyclability, reusable packaging, and the application of innovative barrier materials to replace conventional multilayer materials. A central factor in the design of food packaging is the selection of materials.

The choice of materials for food packaging has been dominated by plastics for decades. These mainly consist of four commodity families, such as PE, PP, PET, and PVC. However, due to their environmental impact, especially at the end of their life, they need to be gradually replaced by more sustainable options. In this respect, bioplastics offer a competitive solution. However, barriers still need to be overcome, such as the high cost and low production yields of biobased feedstocks; their low technical performance, which can be improved by strategies such as compounding and blending, multilayer processing, and coating; their difficult processability due to a greater thermal sensitivity, brittleness, and tackiness; and their biodegradability, which, apart from their release properties, still needs to take into account their blending with other polymers with different biodegradation rates or the availability of appropriate waste management routes.

In terms of processability, the available manufacturing routes for plastic food packaging are still based on the classical ones, such as extrusion, blown film extrusion, injection moulding, or thermoforming. Although extrusion is well established, it still poses challenges in terms of melt flow control, dosage, and thermal stability. As a result, various optimisation methods have emerged based on the decomposition of the processing parameters and the creation of mathematical models. These have improved with the development of computational fluid dynamics and 3D finite element methods.

On the other hand, the introduction of Industry 4.0 and 5.0, with their associated use of networked data and advanced technologies, will be key to the implementation of bioplastics in food packaging and the reduction in associated greenhouse gas emissions. All of this with the use of digital twins can simulate processes and predict potential failures. In addition, machine-aided learning can help to facilitate problem solving, and decision support systems will apply technology to assist in decision making during processing. Developments in intelligent packaging provide tool to support the application of Industry 4.0 and 5.0 in the food packaging sector.

## Figures and Tables

**Table 1:** An overview of the selected winners of the German Packaging Award 2024 and 2023 (where indicated). The images are property of the respective company listed in the table and were released for the German Packaging Award.

Product	Material Type	Advancement	Benefit	Company	Image	Ref.
Injection-moulded tub for dairy products	Polymer (PP)	Reducing overall thickness, vertical bars on inner walls for enhanced stability	20% reduction in material use, 50% increase pot volume per transport box (compared to common market alternatives)	DMK Deutsches Milchkontor GmbH (Zeven, Germany), Pöppelmann GmbH und Co. KG (Lohne, Germany)	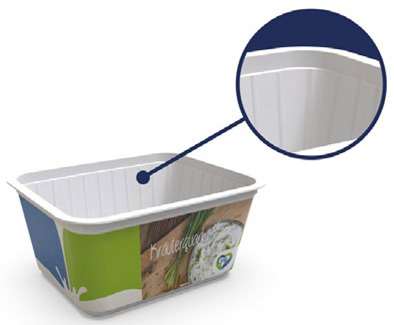	[87]
EcoPeelCover: lidding solution for PP cup materials	Polymer-based compound	Thinner, more resource efficient lidding material	Reduced amount of coating (50%) and aluminium (25%)	Constantia Flexibles International GmbH (Vienna, Austria), Privatmolkerei Bauer GmbH (Wasserburg am Inn, Germany)	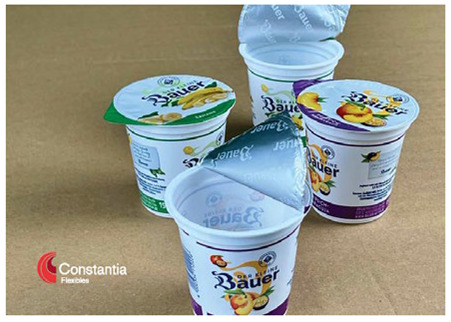	[88,89]
PureWrap PP: wrapper for soft cheese	Polymer (PP)	Woven, fleece-like PP with outer protection layer, providing an air cushion and allowing packaged cheese to mature	Monomaterial replacement for multilayer compounds	LEEB GmbH & Co. KG (Memmingen, Germany)	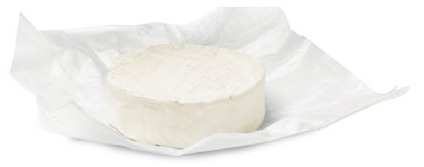	[90]
ShoulderFlex: ultra lightweight bottle (2023)	Polymer (PET)	Bottle with thin walls, stabilised by structural design for durability and stackability	Material reduction of 50%, weight of less than 6 g	Krones AG (Neutraubling, Germany)	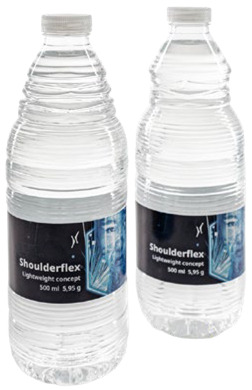	[91]
FlexPod™: pouch alternative to yoghurt cups	Polymer (PE or PP)	Monomaterial pouch for yoghurts	Less material compared to cups, no separation of components, compatible with recycling streams	Wipak Walsrode GmbH (Walsrode, Germany)	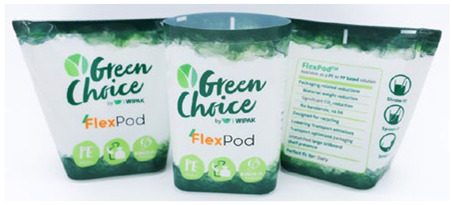	[92]
ReZorce^®^: monomaterial package for beverages	Polymer (HDPE)	Monomaterial alternative to multi-material compounds for beverages	Fully recyclable, high percentage of recycled material (30–70%) due to use of barrier layer, circa 50% reduction in resource and energy use	Zotefoams plc (Croydon, United Kingdom)	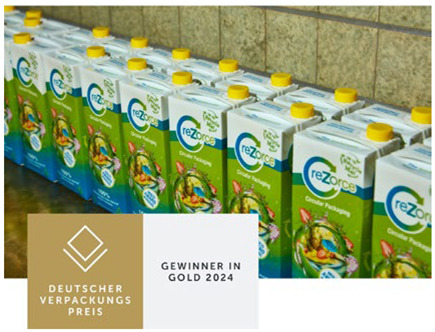	[93]

**Table 2:** Classification of biopolymers depending on their source.

	Main Attribute	Advantages	Disadvantages	General Examples of Implementation	Ref.
Starch-based	Based on starch as raw material or thermoplastic matrix.	Biobased and extracted from several sources.	Material offers poor barrier properties and needs to be blended.	TPS	[104,111]
Cellulose-based	Based on cellulose that is widely available.	Biobased and extracted from several sources.	Hydrophilic with high permeability to water vapour.	Cellulose acetate, cellophane	[104,112]
Aliphatic polyesters	Polyester polymers comparable with conventional plastics.	Improved biodegradability with comparable mechanical properties.	Higher cost and general inferior mechanical performance.	PLA, PHAs, PBS, PCL, PES, PBSA, etc.	[104,113]
Protein-based	Derived from multiple protein sources such as milk or wheat gluten.	Low cost due to their available feedstocks and antimicrobial activity.	Need further enhancement of their mechanical properties and moisture resistance.	Soy, gluten, zein	[104,114]
Chitin-based	Biobased and biodegradable, with widely available biosources.	Natural origin, biodegradability, and good properties	Need further development on its production capacity.	Chitin, PP/chitin	[104,115]

**Table 3:** Proven biodegradation in reference environments for bioplastics, adapted from [107,116].

Bioplastic	Industrial Composting	Home Composting	Anaerobic Digestion	Soil	Fresh Water	Sea Water	Landfill
Cellulose (lignin < 5%)	✔	✔	✔	✔	✔	✔	✔
Cellulose Acetate (and other derivatives)	✔ ^1^	✔ ^1^	✔ ^1^	✔ ^1^	✔ ^1^	✔ ^1^	✔ ^1^
PBAT	✔	✔ ^1^	✖	✔ ^1^	✖	✖	✖
PCL	✔	✔	✖	✖	✖	✖	✖
PBS	✔	✖	✖	✖	✖	✖	✖
PBSA	✔	✔	✖	✔	✖	✖	✖
PHAs ^2^	✔	✔	✔	✔	✔	✔	✔
PLA	✔	✖	✔ ^3^	✖	✖	✖	✖
Starch (and other derivatives)	✔	✔	✔	✔	✔	✔	✔

^1^ For certain grades. ^2^ PHAs including P3HB, P4HB, P3HB4HB, P3HB3HV, P3HB3HV4HV, P3HB3Hx, P3HB3HO, P3HB3HD. ^3^ In thermophilic digestion. Biodegradation conditions are **sea water**: 30 °C, 90% biodegradation within maximum of 6 months; **fresh water**: temperature of 21 °C, 90% biodegradation within maximum of 56 days; **soil**: temperature of 25 °C, 90% biodegradation within a maximum of 2 years; **landfill**: without European standard specifications available; **anaerobic digestion**: thermophilic at 52 °C and mesophilic at 37 °C, 50% biodegradation within 2 months; **home composting**: temperature of 28 °C, 90% biodegradation within maximum of 12 months; **industrial composting**: temperature of 58 °C, 90% biodegradation within maximum of 6 months. PBS: Polybutilsuccinate; PBSA: Polybutilsuccinate adipate.

**Table 4:** Mechanical properties of several variants of PHAs compared to conventional plastics.

	P3HB	P(3HB-co-20%3HV)	P(3HB-co-12%3HH)	P4HB	P(3HB-co-16%4HB)	PP	LDPE
Ref.	[122,124]	[122,124]	[122]	[122,125]	[122]	[124]	[124]
Tm (°C)	177	145	61	60	-	165	120
Tg (°C)	4	−1	−35	−51	-	−10	−100
Cristallinity (%)	50–80	55	-	34	-	50	43
Tensile strength (MPa)	19–40	120	9	50	20	28–40	7–17
Elongation at break (%)	0.8–6	32	380	1000	850	20–75	200–900
